# RNA-binding proteins in ALS and FTD: from pathogenic mechanisms to therapeutic insights

**DOI:** 10.1186/s13024-025-00851-y

**Published:** 2025-06-04

**Authors:** Jens Rummens, Sandrine Da Cruz

**Affiliations:** VIB‐KU Leuven Center for Brain and Disease Research, Department of Neurosciences, KU Leuven, Leuven Brain Institute, Leuven, 3000 Belgium

**Keywords:** Amyotrophic lateral sclerosis (ALS), Frontotemporal dementia (FTD), FTLD, Protein aggregation, Prion-like seeding, Liquid–liquid phase separation (LLPS), RNA-binding proteins (RBP), TDP-43, FUS

## Abstract

Amyotrophic lateral sclerosis (ALS) and frontotemporal dementia (FTD) are devastating neurodegenerative disorders with overlapping clinical, genetic and pathological features. A large body of evidence highlights the critical role of RNA-binding proteins (RBPs) – in particular TAR DNA-binding protein 43 (TDP-43) and Fused in sarcoma (FUS) – in the pathogenesis of these diseases. These RBPs normally regulate various key aspects of RNA metabolism in the nervous system (by assembling into transient biomolecular condensates), but undergo cytoplasmic mislocalization and pathological aggregation in ALS and FTD. Furthermore, emerging evidence suggests that RBP-containing aggregates may propagate through the nervous system in a prion-like manner, driving the progression of these neurodegenerative diseases. In this review, we summarize the genetic and neuropathological findings that establish RBP dysfunction as a central theme in ALS and FTD, and discuss the role of disease-associated RBPs in health and disease. Furthermore, we review emerging evidence regarding the prion-like properties of RBP pathology, and explore the downstream mechanisms that drive neurodegeneration. By unraveling the complex role of RBPs in ALS and FTD, we ultimately aim to provide insights into potential avenues for therapeutic intervention in these incurable disorders.

## Background

Neurodegenerative diseases such as Alzheimer's disease (AD), Parkinson's disease (PD), Huntington's disease (HD), amyotrophic lateral sclerosis (ALS), frontotemporal dementia (FTD) and limbic-predominant age-related TDP-43 encephalopathy (LATE) are characterized by the aggregation of misfolded proteins into insoluble inclusions, which ultimately disrupt cellular function and drive neurotoxicity [1]. Recent studies highlight the prion-like properties of aggregates – where misfolded proteins serve as templates to induce pathological conformational changes in their normal counterparts. This process enables the propagation of protein pathology through the nervous system, which is proposed to drive disease progression [2].

While the prion-like behavior of proteins such as amyloid-β, tau and α-synuclein has been extensively studied in the context of AD and PD, recent evidence suggests that similar mechanisms are at play in ALS and FTD. In these disorders, RNA-binding proteins (RBP) – such as TAR DNA-binding protein 43 (TDP-43) and Fused in sarcoma (FUS) – have been identified as key players in disease pathology. These RBPs assemble into liquid-like biomolecular condensates by a process known as liquid–liquid phase separation (LLPS), which allows them to orchestrate various aspects of RNA metabolism. However, under pathological conditions, they undergo aberrant phase transitions, cytoplasmic mislocalization and aggregation. The prion-like transmission of these aggregates is thought to contribute to the progressive nature of ALS and FTD, making them important targets for therapeutic intervention.

## A brief description of ALS and FTD

Amyotrophic Lateral Sclerosis (ALS) – first described by neurologist Jean-Martin Charcot in 1869 – is a fatal and incurable neurodegenerative disorder that primarily affects the motor system [3–5]. The loss of upper and lower motor neurons in the motor cortex, brainstem and spinal cord ultimately results in paralysis and death typically due to respiratory failure. Most patients experience a rapid disease progression and on average only survive 2–5 years after symptom onset. It is the most common adult motor neuron disease: the cumulative lifetime risk for developing ALS has been estimated at 1:350 for men and 1:400 for women [6]. There is currently no cure or effective treatment for most ALS forms.

The manifestation and progression of ALS symptoms depends on the site of onset. The most common presentation is “spinal onset ALS” in which case the disease starts with muscle weakness in the upper or lower limbs (typically first affecting distal muscles on one side of the body). On the other hand, 30% of patients present with “bulbar onset ALS” where disturbances in speech and swallowing will typically be the first symptoms to arise [7]. Regardless of site of onset, muscle weakness will progressively spread to other (typically adjacent) body parts as the disease advances [8]. Eventually, progressive muscle wasting will culminate in general paralysis with severe swallowing, speaking and breathing difficulties, resulting in the patient’s death [5]. It is important to highlight that the progressive spreading of ALS symptoms to contiguous anatomic regions correlates with the propagation of the underlying neuropathology within the central nervous system (CNS) [8]. Emerging evidence from studies relying on cellular and animal models suggests that this pattern may result from the prion-like aggregation and dissemination of specific misfolded proteins throughout the CNS.

ALS patients (about 35–50%) also present with variable degrees of cognitive and/or behavioral changes, often occurring early in the disease process [9, 10]. About 10–15% of ALS patients receive an additional diagnosis of frontotemporal dementia (FTD) [11]. FTD clinical syndromes include a disorder that is characterized by progressive personality and behavioral deficits known as behavioral variant FTD (bvFTD), and a disorder of speech and language dysfunction known as primary progressive aphasia (PPA) [12]. Each of these FTD syndromes is caused by the progressive degeneration of the frontal and anterior temporal brain lobes – known as frontotemporal lobar degeneration (FTLD) – yet the precise brain region where pathology initiates can vary depending on the clinical picture [12]. FTD is one of the most common forms of early-onset dementia [13–16].

**Fig. 1:**
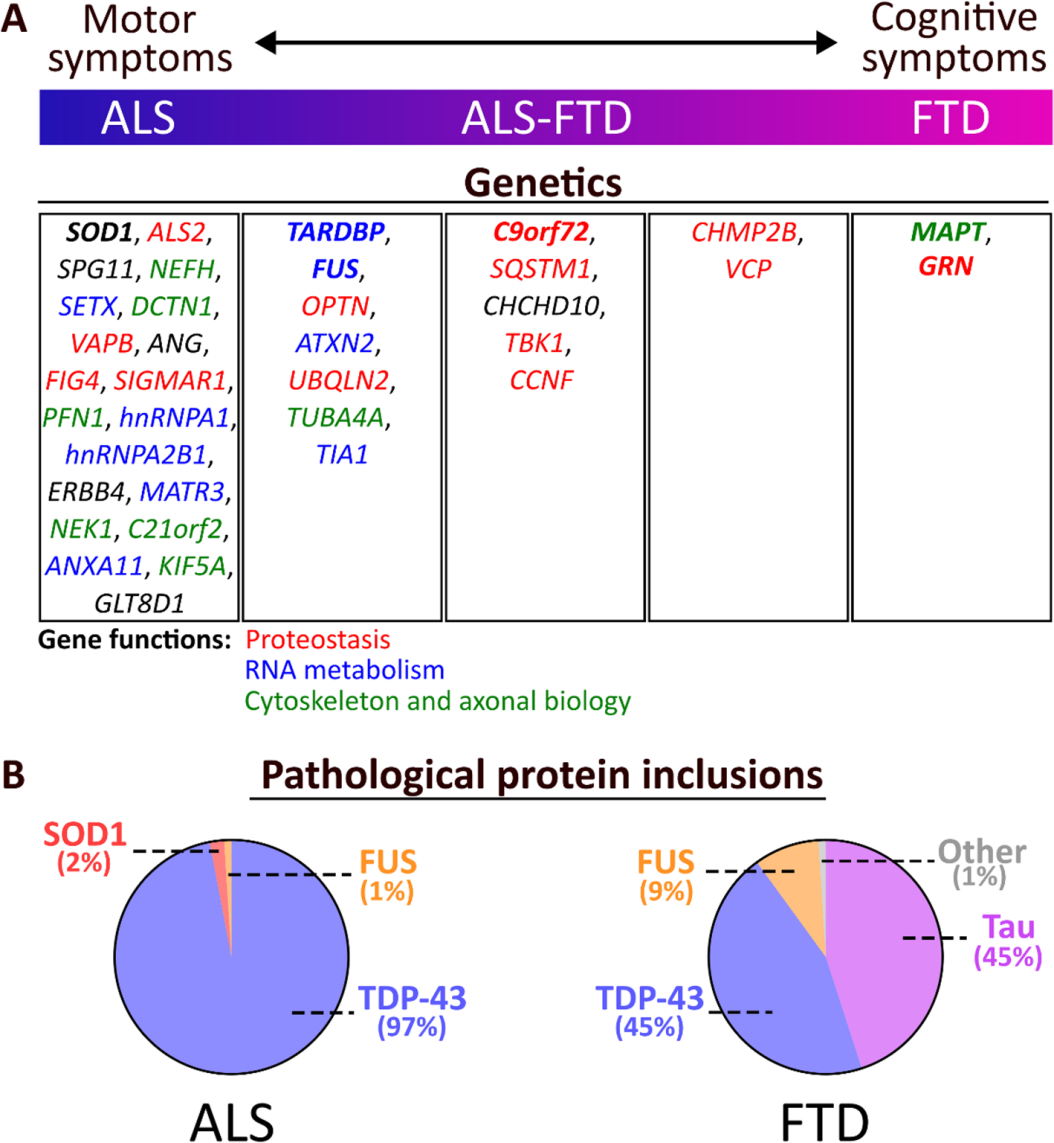
Clinical, genetic and neuropathological overlap of ALS and FTD. **A** ALS and FTD represent two extremes of a broad neurodegenerative disease spectrum. Genetic causes and their relative contribution to ALS (left) and/or FTD (right) are summarized. Colors highlight mechanistic pathways in which these genes have been proposed to function (proteostasis, RNA metabolism, cytoskeleton and axonal biology). Literature sources [17, 18]. **B** Overview of major proteins that accumulate within pathological inclusions in the nervous system of ALS and FTD. Co-pathology of ANXA11 is observed in a subset of FTD patients with TDP-43 pathology (FTLD-TDP type C). Co-pathology of TAF15 (and occasionally EWS) is observed in FTD patients with FUS pathology (FTLD-FUS or FTLD-FET)

The observation that patients with motor neuron disease often exhibit dementia symptoms and vice versa has led to the hypothesis that ALS and FTD represent two ends of a single disease spectrum, commonly referred as “ALS-FTD” [11] (Fig. 1A). This is further supported by recent findings showing ALS and FTD share key genetic and neuropathological features.

### Genetic landscape of ALS and FTD: a central role for RNA binding proteins

ALS can be classified as either familial or sporadic [4]. Familial ALS (10–15%) is by definition transmitted in families and is caused by gene mutations that are typically inherited in a dominant fashion. Mutations in four genes (*C9ORF72*, *SOD1*, *TARDBP* and *FUS*) account for the majority of familial cases. Likewise, 20–25% of FTD patients have a family history with autosomal dominant inheritance. Mutations in three genes (*MAPT*, *GRN* and *C9ORF72*) account for most cases of familial FTD.

Genetic studies in ALS began in 1993 with the seminal discovery of *SOD1* as the first ALS causative gene [19]. Missense mutations in the *SOD1* gene, which encodes the anti-oxidant enzyme superoxide dismutase 1 (SOD1), account for 20% of familial ALS and typically cause disease with high penetrance. Importantly, the mutant SOD1 protein is ubiquitinated and forms insoluble cytoplasmic aggregates in the CNS of patients with ALS-SOD1 [20, 21]. In the following decades, mutations in over 40 additional genes have been reported, albeit several are rare and it remains challenging to unequivocally identify them as the cause of disease [17]. Nonetheless many of these ALS-associated genes cluster in key pathways: (1) proteostasis, (2) RNA metabolism, and (3) cytoskeleton and axonal biology (Fig. 1A).

The *TARDBP* gene encodes TAR DNA-binding protein 43 (TDP-43), a predominantly nuclear DNA/RNA-binding protein which acts as a key regulator of a variety of RNA metabolic functions. Missense mutations in *TARDBP* are responsible for up to 3–5% of familial ALS cases, and have also been identified in sporadic patients [17, 22–25]. In addition, *TARDBP* variants have been associated with different forms of FTD [26, 27]. Most mutations cluster in the aggregation-prone C-terminal domain of the protein [28]. Accordingly, a total of 50 different *TARDBP* mutations in ALS patients, almost all of which heterozygous missense mutations in exon 6 of *TARDBP*, were recently reported [29]. Importantly, four mutations accounted for the majority (51%) of ALS-TARDBP patients (M337 V, A382 T, G294 V, G298S), while the other variants were only identified in a limited number of individuals [29]. Future studies with larger cohorts of patients will therefore be important to definitively confirm the pathogenicity of rare *TARDBP* variants.

Mutations in *fused in sarcoma* (*FUS*) account for 2–8% of familial ALS patients and are typically associated with a severe and rapid disease progression [30–32]. More than fifty ALS-linked *FUS* mutations have been identified [32], most of which cluster in its nuclear localization signal (NLS) [33–35]. Like TDP-43, FUS protein interacts with RNA/DNA and has been associated with a variety of functions in RNA metabolism [36, 37]. While FUS is predominantly present in the nucleus in normal conditions, disease mutations induce its cytoplasmic mislocalization and aggregation in ALS-FUS [30, 31, 36, 38].

Repeat expansion mutations in *C9ORF72* – chromosome 9 open reading frame (ORF) locus 72 – account for 40% of familial ALS cases, as well as 37.5% of familial FTD cases, making it the most common genetic cause of ALS and ALS-FTD [4]. The *C9ORF72* gene harbors an intronic GGGGCC hexanucleotide sequence that abnormally repeats up to hundreds or thousands of times in patients with ALS, FTD or both [39, 40]. Several mechanisms through which this hexanucleotide repeat expansion can drive neurodegeneration have been proposed [41]: 1) loss of the physiological function of the C9ORF72 protein [42], 2) gain of toxicity mediated by either RNA foci, produced from the *C9ORF72* hexanucleotide repeat expansion, forming secondary structures which may aberrantly sequester RBPs [41, 43], and/or the aberrant accumulation of dipeptide repeats (DPR) produced through non-conventional Repeat Associated Non-AUG (RAN) translation of the GGGGCC repeats [41, 44], or 3) both loss and gain of toxicity [45, 46].

Rare ALS and/or FTD associated mutations have also been identified in a number of other genes linked to RNA metabolism. Examples include *hnRNPA1* [47], *hnRNPA2B1* [47], *MATR3* [48–52], *ANXA11* [53–57], *SETX* [58] and *TIA1* [59–61]. In addition, intermediate-length CAG nucleotide repeat expansions in the *ATXN2* gene [62, 63] – which encodes the RBP ataxin-2 [64] – are associated with an increased risk for developing ALS. Altogether, these studies highlight dysfunction and abnormal accumulation of RBPs as a recurring theme in ALS and FTD pathogenesis.

### Aggregation of RNA-binding proteins in ALS and FTD

Aggregation of misfolded proteins is a hallmark of most neurodegenerative disorders [1]. In ALS, the most common pathology involves aggregation of the RBP TDP-43 (ALS-TDP), found in approximately 97% of sporadic and familial ALS cases. The remaining ALS cases are characterized by SOD1 aggregation (ALS-SOD1, ± 2%) or FUS inclusions (ALS-FUS, < 1%), linked to mutations in the respective genes. In FTD, the most common pathology is the aggregation of either TDP-43 (FTLD-TDP, ± 45%) or microtubule-associated tau protein (FTLD-tau, ± 45%). Finally about 9% of the cases are characterized by aggregates of wild-type FUS, originally defined as FTLD-FUS, but more recently also known as FTLD-FET, due to the co-aggregation of the related RBPs Ewing’s sarcoma protein (EWS) and TATA-binding protein-associated factor 15 (TAF15), all three define the FET family [65, 66]. These different subtypes underscore the molecular heterogeneity of ALS and FTD, and provide a framework for understanding the overlapping pathological mechanisms driving these diseases [11] (Fig. 1B).

#### TDP-43 pathology as a hallmark of ALS, FTD and other diseases

Detergent-insoluble cytoplasmic inclusions that are ubiquitin-positive, but negative for tau and α-synuclein, are widely recognized to accumulate in the CNS of ALS and a subset of FTD patients [67, 68]. In 2006, a landmark study by Neumann and colleagues identified TDP-43 as the main constituent of these insoluble inclusions using an antibody-based approach combined with mass spectrometry [69]. Pathological TDP-43 in patient-derived aggregates is ubiquitinated, hyperphosphorylated and (in some instances) C-terminally truncated. Cytoplasmic aggregation of TDP-43 in affected cells is frequently associated with its depletion from the nucleus [69, 70]. Together, abnormal nuclear depletion and cytoplasmic aggregation of TDP-43 are the key TDP-43 pathological hallmarks. Interestingly, although less prevalent, TDP-43 inclusions have also been reported inside nuclei of affected neurons and glia [69, 70]. It is noteworthy that C-terminal truncation of TDP-43 is commonly associated with TDP-43 pathology in the brain, but rarely observed in the spinal cord of individuals with ALS and FTD, suggesting that the molecular composition of pathological TDP-43 inclusions is (to some extent) tissue-specific [71, 72]. As discussed previously, pathogenic mutations in *TARDBP* were subsequently identified as a genetic cause of ALS [22, 23], yet the majority of ALS patients with TDP-43 pathology do not harbor *TARDBP* mutations. This suggests that ALS-causing genetic mutations and pathogenic mechanisms eventually converge into TDP-43 cytoplasmic mislocalization/aggregation.

In 2013, Brettschneider and colleagues analyzed a cohort of 76 clinically-confirmed ALS autopsied cases to map the distribution of phosphorylated TDP-43 pathology throughout the nervous system, resulting in the identification of four distinct disease stages [73]. In ALS cases with the lowest pathological burden, TDP-43 aggregation was primarily confined to motor neurons of the agranular motor cortex, brainstem and spinal cord. However, with increasing pathology burden, phosphorylated TDP-43 accumulation progressed from the sites of initiation to connected CNS regions in a stereotypical manner [73]. This is reminiscent of reported prion-like spreading of disease proteins such as tau and α-synuclein in other neurodegenerative diseases [2]. The distribution of phosphorylated TDP-43 pathology has further been reported to correlate with neuronal loss in ALS spinal cord and brain autopsies [74], suggesting that the spreading of TDP-43 pathology may contribute to the progressive neurodegeneration and dissemination of muscle wasting in ALS patients [8]. Furthermore, TDP-43 aggregates in ALS display significant morphological heterogeneity, and a recent investigation of post-mortem brain autopsies from 61 ALS cases (without FTD) proposed that ALS-TDP can be further classified in three histopathological subtypes – denoted as ALS-TDP type E, type B and type SC (scarce cortical) – based on the morphology, abundance and composition (i.e. colocalization with p62) of phosphorylated TDP-43 pathology in the motor cortex [75]. Specifically, the defining pathological features of ALS-TDP type E are abundant pTDP-43 granulofilamentous inclusions which are p62-negative, ALS-TDP type B on the other hand is characterized by neuronal cytoplasmic inclusions which are immuno-positive for both pTDP-43 and p62, while ALS-TDP type SC has scarce cortical pTDP-43 and p62 aggregates. No differences in age at death or disease duration could be detected. More research will be needed to further determine whether these newly proposed pathological subtypes are associated with unique clinical phenotypes or different stages of pathology as proposed by Brettschneider and colleagues [73].

While most neuropathological studies on TDP-43 pathology focus on the CNS, phosphorylated TDP-43 accumulation has recently been described in peripheral nerves and muscles from ALS patients [76], as well as multiple systemic tissues including the gastro-intestinal tract, lymph nodes and blood vessels [77]. Interestingly, this pathology is detected prior to onset of ALS symptoms, potentially offering valuable diagnostic opportunities. Further research is eagerly anticipated to characterize in more depth the presence of TDP-43 aggregation outside the CNS, and ultimately determine its role in disease onset and progression.

TDP-43 pathology is also a feature of ± 45% of patients with FTLD, with or without concomitant motor neuron disease [12, 69, 78]. The morphology and distribution of TDP-43 aggregates in FTLD-TDP is heterogeneous, which serves as the basis for the classification of FLTD-TDP neuropathological subtypes [79–81]. FTLD-TDP type A is marked by oval or crescentic shaped neuronal cytoplasmic inclusions and dystrophic neurites in the superficial cortical layers, and occasional intranuclear neuronal inclusions. This subtype is found in all familial FTD patients with mutations in *GRN* and a subset of patients with *C9ORF72* mutations [79]. FTLD-TDP type B pathology typically presents as diffuse or granular TDP-43-containing neuronal cytoplasmic inclusions across all cortical layers. It is often associated with *C9ORF72* mutations and is common in cases where FTLD co-occurs with ALS [79]. In contrast, FTLD-TDP type C cases display few cytoplasmic inclusions, with abundant dystrophic neurites, primarily in superficial cortical layers. No genetic associations have been reported [79]. These three subtypes (FTLD-TDP type A-C) account for the majority of FTLD patients (81–96%) [82]. More recently, two novel and less common pathology subtypes have been described (FTLD-TDP type D and E). FTLD-TDP type D is exclusively found in patients with mutations in the *VCP* gene, characterized by abundant intranuclear neuronal inclusions and short dystrophic neurites [79]. FTLD-TDP type E is described to be mostly similar to FTLD-TDP type B and is defined by the unique presence of granulofilamentous neuronal cytoplasmic inclusions in all cortical layers that are often negative for ubiquitin [83]. Although the origin of this pathological heterogeneity is unclear, TDP-43 assemblies extracted from different subtypes are emerging to display distinct biochemical properties and amyloid-like structures [81, 84–86], and are linked to unique clinical presentations [79, 80].

TDP-43 pathology in FTLD – similar to ALS – typically encompasses both cytoplasmic aggregation and nuclear depletion of TDP-43 in affected cells [12, 69, 87]. Interestingly, a recent autopsy study examining TDP-43 pathology in von Economo neurons and fork cells of the frontoinsular cortex, the most vulnerable cell types in bvFTD [88], reported that a minority of these neurons display nuclear depletion of TDP-43 without detectable inclusions [87]. It is not clear whether this suggests that nuclear depletion of TDP-43 precedes the formation of cytoplasmic aggregates or that TDP-43 aggregates were cleared in these neurons, or alternatively, that TDP-43 aggregates remained undetected in these cells due to technical limitations associated with visualizing TDP-43 aggregates in autopsied samples. Further work is thus needed to better understand these neuropathological observations in end-stage CNS. Furthermore, phosphorylated TDP-43 aggregation in a cohort of FTLD-TDP autopsy cases with bvFTD (*n* = 39) was also reported to spread sequentially from the site of initiation (orbital gyri, gyrus rectus, and amygdala) to connected brain regions as disease pathology progresses [89], further supporting a prion-like propagation mechanism of TDP-43 pathology.

It is important to highlight that TDP-43 pathology in glia – particularly oligodendrocytes – is also commonly described in ALS and FTLD [73, 89, 90], and glial cells have been proposed to play an important role in disease progression [91]. Fatima and colleagues evaluated the distribution of phosphorylated TDP-43 inclusions in oligodendrocytes across the CNS of post-mortem ALS cases by immunohistochemistry (*N* = 34), and found abundant oligodendrocyte phosphorylated TDP-43 pathology in affected grey matter regions – but not in connecting deep white matter tracts [92]. Hence whether or not oligodendrocytes are involved in propagation of TDP-43 pathology between neurons remains unclear. Experimental studies will be needed to directly investigate this question.

Over the past decade, TDP-43 aggregation has also been associated with an increasing number of other neurodegenerative diseases. Collectively, disorders with TDP-43 pathology are termed “TDP-43 proteinopathies”. A recently identified example is “limbic-predominant age-related TDP-43 encephalopathy” (LATE), a dementia syndrome clinically similar to AD typically affecting individuals over 80 years of age [93, 94]. LATE is characterized by cytoplasmic aggregation of phosphorylated TDP-43 in neurons and glia of the limbic system, which is often (but not always) accompanied by hippocampal sclerosis in advanced cases. TDP-43 pathology is confined to the amygdala in the initial stage of LATE, and typically spreads to the hippocampus and middle frontal gyrus – and sometimes also regions outside the limbic system – as the disease progresses [93, 94].

In addition to its involvement in primary TDP-43 proteinopathies, TDP-43 aggregation is recognized as a secondary pathology in neurodegenerative disorders such as AD [95], HD [96] or Lewy body disease [97] – as well as systemic diseases like inclusion body myopathy (IBM) [98, 99] – where TDP-43 pathology can occur alongside aggregation of other disease proteins [100]. Despite the underlying mechanisms being unknown, mounting evidence suggests that TDP-43 pathology may also contribute to the pathogenesis of these disorders. For instance, TDP-43 inclusions were immunodetected in the majority of AD cases (up to 57%) [95] – in some instances in neurons with neurofibrillary tangles [101]. Interestingly, multiple reports describe more severe cognitive impairments in AD patients with TDP-43 co-aggregation [95, 101–104]. More recently several independent studies further confirmed nuclear clearance and loss-of-function of TDP-43 in the CNS of AD patients [105–107]. TDP-43 aggregation is also a common feature in HD, where TDP-43 has been shown to be incorporated in mutant huntingtin (HTT)-containing pathological inclusions [108]. Interestingly, mutant HTT was recently reported to cause TDP-43 loss-of-function – along with altered N6-methyladenosine (m6 A) RNA modifications – which in turn drive RNA splicing abnormalities in HD [96]. A more detailed description of TDP-43 pathology in other TDP-43 proteinopathies is beyond the scope of this manuscript, and can be found elsewhere [100].

#### Other RBPs in TDP-43 proteinopathies

While TDP-43 proteinopathies are by definition characterized by pathology of TDP-43, a number of other RBPs have been reported to form aggregates in specific subsets of patients as well. For instance, mutations in the *MATR3* gene – which encodes RBP Matrin 3 – account for ± 1% of ALS cases [48–52]. Matrin 3 has been identified as a component of neuronal cytoplasmic inclusions in motor neurons of a number of sporadic ALS cases [109] and cases with *C9ORF72* mutations [48, 110], although more research is needed to unambiguously confirm Matrin-3 aggregation as a pathological feature of ALS [111, 112]. Rare mutations in the related heterogeneous nuclear ribonucleoproteins hnRNPA1 and hnRNPA2B1 have also been reported in a small subset of patients with ALS as well as a rare disorder termed multisystemic proteinopathy (MSP) – which is characterized by progressive degeneration of muscle, brain, motor neurons and bone, in combination with pronounced TDP-43 pathology [47]. Both hnRNPA1 and hnRNPA2B1 display nuclear depletion and cytoplasmic accumulation in muscle biopsies of MSP patients, and partially co-localize with cytoplasmic TDP-43 inclusions [47]. Another target which appears of particular importance in ALS and FTLD-TDP is annexin A11, a phosphoinositide binding protein which acts as a molecular tether between RNA granules and lysosomes to facilitate their transport along neuronal axons [113]. Rare mutations in the annexin A11 gene (ANXA11) may cause ALS with or without FTLD [53–57], and abnormal annexin A11 aggregates that occasionally co-localize with TDP-43 inclusions have been reported in brain/spinal cord autopsies from ALS cases with ANXA11 mutations [54, 57]. Interestingly, recent immunohistochemical examination of 368 autopsy cases reported co-pathology of annexin A11 and TDP-43 in all sporadic FTLD-TDP type C cases – as well as a small proportion (3–6%) of FTLD-TDP type A-B, ALS and LATE cases – suggesting that combined pathology of annexin A11 and TDP-43 also occurs in the absence of *ANXA11* mutations [114]. Elegant work from Arseni and colleagues revealed that annexin A11 co-assembles with TDP-43 in amyloid-like filaments extracted from FTLD-TDP type C patient brains, as detailed below [86]. Interestingly, an ANXA11 variant (P93S) has recently also been linked with corticobasal degeneration, and expression of this pathogenic ANXA11 variant reportedly induced TDP-43 mislocalization and dysfunction in cultured iPSC-derived neurons [115]. Altogether these findings suggest an important role of annexin A11 in TDP-43 proteinopathies, and highlight the need for further research into aggregation-prone RBPs. Exciting advancements in single-cell mass spectrometry have recently revealed a proteomic signature of individual human ALS motor neurons captured by laser micro-dissection from *postmortem* tissues [116]. The widespread application of such technologies will be invaluable in providing deeper insights into the heterogeneous composition of TDP-43 aggregates in human diseases.

#### FUS pathology in ALS and FTD

FUS pathology in ALS is exclusively observed in the subset of familial ALS cases with mutations in the *FUS* gene [30, 31, 117]. Most of these mutations disrupt or delete the nuclear localization signal, impairing nuclear import and causing cytoplasmic mislocalization of the FUS protein [33–35]. ALS-FUS cases are marked by aberrant accumulation of cytoplasmic FUS inclusions in neurons and glia, some of which are reported to be immunoreactive for ubiquitin–proteasome system components (such as ubiquitin and p62), and are often accompanied by reduced nuclear FUS. Instead, TDP-43 pathology is consistently absent from ALS-FUS [30, 31, 118–124]. It is noteworthy that extensive variability in FUS pathology has been observed: a comparative analysis of six ALS-FUS cases reported different shapes of neuronal cytoplasmic FUS inclusions (round versus tangle-like) depending on disease severity and mutation [125]. Moreover, there was significant heterogeneity in the anatomic distribution of FUS pathology in the CNS, although FUS inclusions in the lower motor neurons were consistently observed in all cases [125]. Nonetheless, efforts to map the distribution of FUS pathology in ALS have been hampered by the rarity of *FUS* mutations. Post-mortem studies with larger sample sizes will be invaluable to provide more insight into the spreading patterns of FUS pathology in ALS.

FUS cytoplasmic inclusions are also a feature of FTLD cases without tau or TDP-43 pathology (FTLD-FUS or FTLD-FET) (± 9%) [126–129] – historically referred to as atypical FTLD-U, neuronal intermediate filament inclusion disease (NIFID) and basophilic inclusion body disease (BIBD) [128, 130]. Importantly, genetic analyses of pathologically confirmed FTLD-FUS cases have not identified any mutations in the *FUS* gene [126–129], which is in sharp contrast with ALS-FUS where *FUS* mutations are present in all cases. Hence, FUS aggregates in FTLD appear to be formed by wild-type rather than mutant FUS.

Besides neuronal FUS pathology in ALS and FTLD, FUS-immunoreactive inclusions have been reported to accumulate in glial cells (particularly oligodendrocytes) as well [117, 119, 131]. How glia contribute to the propagation of FUS pathology in the nervous system remains poorly understood.

While cytoplasmic aggregation of FUS has so far only been linked with the pathogenesis of ALS and FTD, a number of immunohistochemical and mass spectrometry-based studies have also reported FUS in intranuclear neuronal inclusions that accumulate in polyglutamine repeat expansion disorders, including HD, spinocerebellar ataxia type 1 and 3 (SCA 1 and SCA 3), and a rare multisystem neurodegenerative condition termed neuronal intranuclear inclusion body disease (NIIBD) [132–135]. Further research is thus needed to elucidate the potential role of these intranuclear FUS inclusions in the pathogenesis of polyglutamine disorders.

#### Other RBPs in FUS proteinopathies

As outlined above, FUS belongs to the so-called FET protein family that also includes the ubiquitously expressed RBPs EWS and TAF15 [117]. Recombinant FUS, EWS and TAF15 proteins were found to physically interact and form homo- or heterotypic protein complexes [136]. Importantly, several reports describe co-recruitment of TAF15 to FUS-positive cytoplasmic inclusions in FTLD-FUS/FLTD-FET patients, with reduced TAF15 levels in the nuclei of aggregate-containing cells [65, 66]. EWS has also been reported to co-accumulate in FTLD-FUS/FTLD-FET aggregates albeit with more variable mislocalization [65, 66]. In contrast, FUS aggregates in the CNS of ALS patients are not immunoreactive for EWS or TAF15, highlighting the distinct molecular composition of pathological FUS inclusions in ALS-FUS versus FTLD-FUS/FTLD-FET [66]. This is further supported by recruitment of the cargo transport protein transportin-1 (also named Importin-β2, which mediates nuclear import of FET proteins) to FUS/EWS/TAF15-containing pathological inclusions in FTLD, but not FUS-containing inclusions in ALS [65, 137, 138]. Given this, it is plausible that the cytoplasmic mislocalization and aggregation of FUS, along with EWS and TAF15, results from defective nuclear import of FET proteins in FTLD patients. Surprisingly, a recent structural study identified TAF15 – rather than FUS – as the main component of amyloid filaments extracted from four brains of FTLD patients with FUS/TAF15 pathology [139]. These findings suggest a potential role of TAF15 in the pathogenesis of FTLD, previously underestimated, and emphasizes the need to further understand the relationship between the three members of the FET family in disease.

## Structure and functions of TDP-43 and FUS

TDP-43 and FUS are ubiquitously expressed and evolutionary conserved DNA/RNA-binding proteins, that belong to the hnRNP family and are involved in a myriad of cellular processes – mostly related to RNA metabolism [140, 141]. While TDP-43 and FUS predominantly reside in the nucleus under physiological conditions, they also fulfill critical functions in the cytoplasm [37, 142] and have been shown to shuttle between both compartments [38, 143–145].

**Fig. 2:**
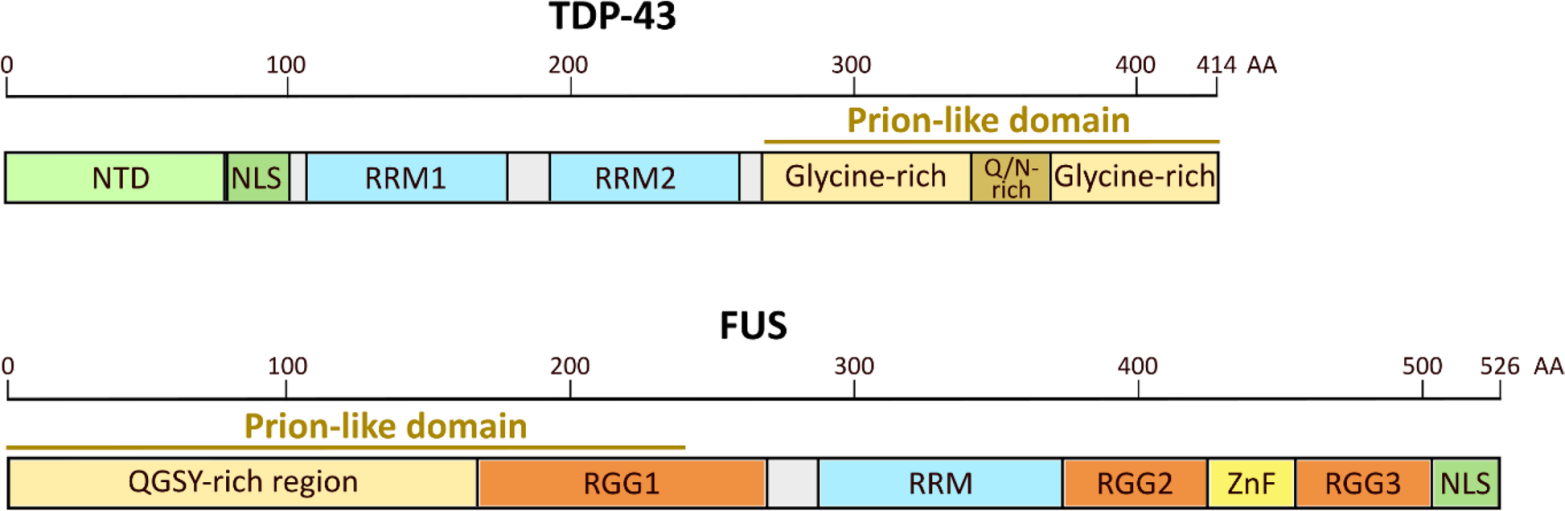
Structure of TDP-43 and FUS. N-terminal domain (NTD); RNA-recognition motif (RRM); NLS (nuclear localization signal); Zinc finger domain (ZnF). In addition to the N-terminal prion-like domain of FUS, a small prion-like region might reside within FUS RGG2 (not indicated on schematic) [160, 161]

TDP-43 comprises 414 amino acids and consists of an N-terminal domain with a classical NLS, two RNA-recognition motifs, and a C-terminal domain which is largely disordered – also termed low-complexity domain [146] (Fig. 2). The TDP-43 N-terminal domain (NTD) is stably folded in a ubiquitin-like or DIX-domain-like structure [147–149], and has been proposed to facilitate TDP-43 assembly into functional dimers or oligomers [149–153]. NTD-mediated dimerization/oligomerization of TDP-43 is required to support its physiological functions including RNA splicing [149–153], and potentially acts to antagonize pathological TDP-43 aggregation by spatially separating aggregation-prone C-terminal domains of consecutive TDP-43 monomers [149]. The NLS of TDP-43 is recognized by the importin-α/β1 heterodimer to facilitate its active transport into the nucleus [154]. Hence, mutation or deletion of the NLS induces TDP-43 misaccumulation in the cytoplasm [155]. While the existence of a nuclear export signal (NES), potentially enabling TDP-43 shuttling from the nucleus to the cytoplasm [143, 155] had previously been reported, this has been contradicted by more recent findings [144, 145, 156]. The current consensus view proposes that TDP-43 export from the nucleus occurs primarily via passive diffusion. TDP-43 also contains two highly conserved RNA-recognition motifs (RRM1 and RRM2), each composed of five β-strands and two α-helices [157], which are critical to mediate interaction with UG-rich RNA sequences [157–159]. Multiple studies demonstrated that TDP-43 interaction with RNA [144, 153, 156] – as well as its oligomerization behavior [153] – are essential to preserve its nuclear localization through formation of macromolecular complexes that slow down diffusion into the cytoplasm.

The C-terminus of TDP-43 harbors an intrinsically disordered low-complexity domain (LCD) which comprises a glycine-rich region as well as a segment that is enriched in glutamine and asparagine residues [146]. This LCD is also referred to as prion-like domain, owing to its resemblance to prion proteins in yeast [162, 163]. The TDP-43 LCD has the ability to de-mix into dynamic condensates via a process called liquid–liquid phase separation (LLPS), which is mediated in part by a conserved sequence within this domain that can transiently fold into a α-helical structure [164, 165]. Hence TDP-43 LLPS has been proposed to tune TDP-43 binding to RNA targets and affect its roles in RNA processing [165–167]. The prion-like domain of TDP-43 is also particularly important for its pathological behavior. Firstly, the TDP-43 LCD is highly aggregation-prone, both in vitro and in cells [168–170]. Secondly, the majority of ALS-causing *TARDBP* mutations and phosphorylation sites reside in the TDP-43 LCD [28]. Thirdly, C-terminal TDP-43 fragments of ∼25–35 kDa are frequently found inside insoluble inclusion bodies extracted from autopsied material with TDP-43 proteinopathy, and are proposed to contribute to toxicity [71]. It is noteworthy that alternative isoforms that lack the most C-terminal region of TDP-43, termed short TDP-43, were recently reported to be upregulated in human iPSC-derived neurons specifically in response to neuronal hyperactivity, which was associated with neuronal toxicity [171].

FUS comprises 526 amino acids and shares a number of structural features with TDP-43 [160] (Fig. 2). FUS (“Fused in sarcoma”) was initially identified as part of a chromosomal translocation in human liposarcomas where the *FUS* gene is fused to the gene *CHOP*, whereby the RNA-binding domains of FUS are replaced by the basic leucine zipper domain of CHOP [172]. At its N-terminus, FUS harbors a prion-like and aggregation-prone LCD that is enriched in glutamine, glycine, serine and tyrosine residues (QGSY region). It also contains multiple nucleic acid binding domains that facilitate its RNA regulatory functions, including a single RRM, three arginine-glycine-glycine repeat regions (RGG1-3) and a zinc-finger motif (ZnF) [160]. Multivalent interactions among tyrosine residues in the prion-like QGSY region and arginine residues within the RNA-binding domains, respectively, enable FUS to undergo LLPS [173–175]. At its C-terminal end, FUS harbors a non-classical proline-tyrosine nuclear localization signal (PY-NLS), which is recognized by transportin 1 for its nuclear import [176]. This latter process is impaired in FUS-mediated neurodegenerative diseases. Indeed, the majority of ALS-causing *FUS* mutations reside in the NLS motif, and several reports have demonstrated that these mutations affect the interaction between FUS and transportin 1, ultimately causing cytoplasmic FUS misaccumulation [33–35]. Finally, pathological cytoplasmic FUS in the context of FTLD – but not ALS – is reportedly associated with reduced methylation of FUS at the RGG3 domain (next to the PY-NLS) [177], and this hypomethylation influences the interaction between FUS and transportin 1 – which could in turn contribute to abnormal cytoplasmic FUS accumulation [177].

### Role in RNA metabolism

RNA immunoprecipitation-based approaches such as CLIP-seq (crosslinking and immunoprecipitation sequencing) have been invaluable to map RNA interactomes of TDP-43, whereby TDP-43 is reported to bind more than 6000 mRNA targets, preferentially within UG-rich RNA stretches [178–181]. Hence TDP-43 is thought to regulate a considerable fraction of the transcriptome. This is supported by a wealth of research relying on RNA sequencing efforts performed in human and mouse systems upon TDP-43 depletion including cultured cells with genetic silencing of TDP-43 (such as human iPSC-derived neurons [182, 183], neuron-like SH-SY5Y cells [184, 185] and non-neuronal cell lines [186]; mouse primary neurons [187], neuron-like and muscle cell lines [188]), sorted neuronal nuclei with TDP-43 nuclear clearance isolated from post-mortem human ALS-FTD brains [189], or mouse brain tissue following antisense oligonucleotide (ASO)-mediated TDP-43 knockdown [178]. Altogether loss of TDP-43 results in widespread transcriptomic changes, which include profound alterations in expression and splicing of numerous essential genes [178, 182, 184, 186, 190–194].

Likewise, FUS is reported to bind to more than 5500 RNAs, preferentially within long introns, in human cell lines, cultured neurons, mouse brain and human post-mortem brain tissue [195–198]. FUS is reported to bind with limited sequence specificity to GGU and related RNA motifs [195, 196], although such motifs appear neither necessary nor sufficient for FUS-RNA interaction [199]. Genetic silencing of FUS also results in vast changes in mRNA splicing and gene expression levels, i.e. FUS depletion in the adult mouse brain altered the levels or splicing of about 950 mRNAs [196]. Yet the transcripts affected by TDP-43 and FUS are largely distinct [195, 196]. The small subset of overlapping RNAs regulated by both TDP-43 and FUS comprise long-intron containing RNA transcripts that are essential for neuronal development and integrity. This converging pathway may be highly relevant in both TDP-43-mediated and FUS-mediated neurodegenerative diseases [195, 196].

A limited number of studies have evaluated RNA interactomes of the other members of the FET protein family, revealing that EWS and TAF15 have largely similar binding profiles to FUS – albeit no major effect on alternative RNA splicing (unlike FUS and TDP-43) [198, 200].

**Fig. 3:**
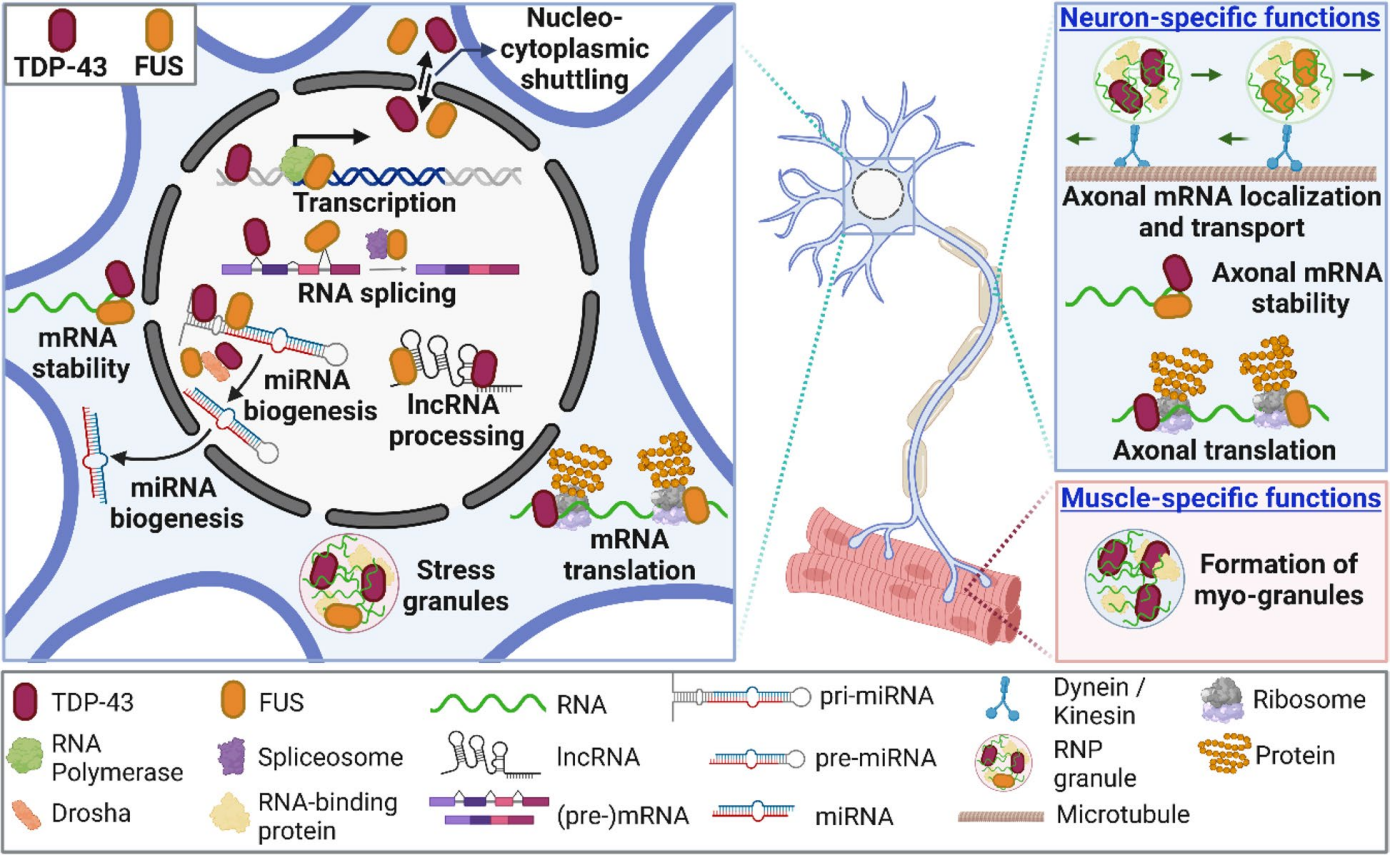
Physiological functions of TDP-43 and FUS as regulators of RNA metabolism in a cell- and compartment-specific manner

In light of the crucial roles of TDP-43 and FUS in the regulation of RNA metabolism [159], their dysregulation (in model systems or disease) is associated with defects in several steps of RNA processing, including transcription [201–204], splicing [195, 197, 205], RNA stability [206, 207], mRNA transport [37, 208, 209], translation [37, 142, 210], stress granule formation [211], and processing of miRNAs and lncRNAs [212–215] (Fig. 3). Accordingly, proteomic studies revealed protein interactomes of ectopically-expressed TDP-43 [216, 217] and FUS [218] in cultured cells which include numerous other RNA metabolic-related proteins, such as components of RNA splicing, translation and miRNA processing machinery. TDP-43 and FUS were also found to interact with each other, and this was more pronounced in the presence of ALS-linked TDP-43 mutations [216]. Interestingly, TDP-43 also regulates stability of its own *TARDBP* transcript by interacting with the 3’UTR – thereby tightly regulating its own expression level through a negative feedback loop [178, 179, 219]. A similar autoregulation mechanism has been proposed for FUS, whereby FUS binds and affects splicing of its own mRNA with retention of introns 6 and 7 [196, 220, 221]. The existence of such autoregulatory feedback mechanisms suggests that a tight control of TDP-43 and FUS expression is important for cellular homeostasis. It is noteworthy that TDP-43 and FUS also participate in cellular processes that are not directly related to RNA metabolism, including DNA repair and regulation of genome integrity, which have been comprehensively reviewed elsewhere [222].

Besides their functions in soma, emerging evidence also highlights an important role for both TDP-43 and FUS in the local regulation of RNA metabolism in axons and synapses of neurons [37], such as axonal mRNA stability and transport [208, 209, 223] and local translation at motor axons and neuromuscular junctions (NMJs) [210, 224, 225] (Fig. 3). Importantly, these compartment-specific RNA regulatory functions are emerging to be impaired in disease and to contribute to the (early) stages of ALS pathogenesis [37]. Furthermore, it is important to highlight that TDP-43 and FUS are ubiquitously expressed proteins and also fulfill essential RNA regulatory functions outside the nervous system such as muscle tissue (Fig. 3). Indeed TDP-43 depletion in muscle cells results in widespread mRNA dysregulation which is distinct from neuronal cells [188]. Interestingly, TDP-43 assembles into RNA-containing myo-granules that are essential for skeletal muscle development and regeneration [226]. A role for FUS as a transcriptional regulator in muscle cells at the NMJ where it is enriched in subsynaptic myo-nuclei and regulates transcription of the acetylcholine receptor gene has also been described. This function is found to be compromised in an ALS-FUS mouse model and iPSC-derived systems [227]. Further work is needed to fully elucidate how TDP-43 and FUS regulate RNA metabolism in distal neuronal compartments and non-neuronal cell types such as muscle cells.

### Liquid–liquid phase separation

Liquid–liquid phase separation (LLPS) describes a process in which a homogeneous mixture of molecules separates (“de-mixes”) into two co-existing liquid phases: a dilute phase and a condensed phase [228–230]. A classic example of LLPS outside the field of biology is the separation of oil and water, in which oil de-mixes from the water solution to form droplets (essentially generating a liquid within a liquid). The same biophysical principle enables cells to sequester biomolecules in membraneless liquid-like organelles – also termed biomolecular condensates. This concept traces back to pioneering observations by cell biologist Edward B. Wilson, who first proposed the droplet-like behavior of cytoplasmic structures inside the cell in 1899 [231]. However, it is only more recently that researchers have begun to realize that LLPS is a general mechanism underlying the formation of various membraneless organelles in the cell. In a seminal 2009 study, Hyman and Brangwynne found that P granules (a type of RNA- and protein-containing organelle in embryos of *Caenorhabditis elegans*) display liquid-like properties: they are dynamic and reversible, have a spherical shape, and can undergo fusion/fission [232]. Since then, several other membraneless organelles with liquid-like properties have been reported to govern a wide range of biological processes throughout the cell. Examples include the nucleolus [233–235], centrosome [236], synaptic density [237–241] and various types of RNA bodies [229, 242].

**Fig. 4:**
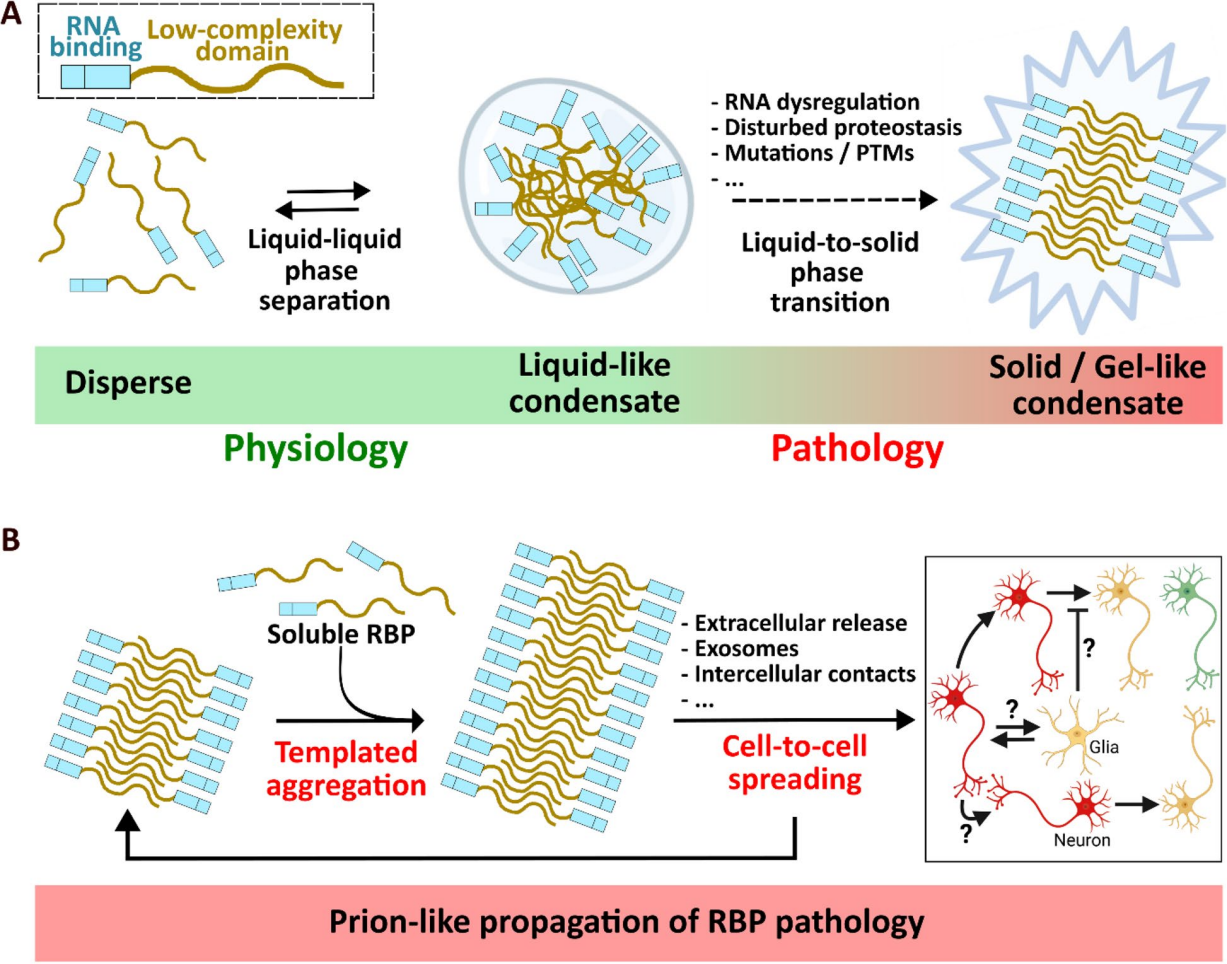
RNA-binding protein (RBP) condensation, aggregation and spreading. **A** Phase separation of RBPs in physiology and neurodegenerative disease. Under physiological conditions, RBPs de-mix into transient liquid-like condensates via LLPS. Several pathological events can trigger aberrant phase transitions that result in the formation of irreversible solid/gel-like assemblies. Post-translational modifications (PTMs). **B** Schematic representation of prion-like hypothesis for RBPs. Formation of an amyloid-like RBP aggregate triggers templated amplification and spreading of RBP pathology in the nervous system

An important subset of biomolecular condensates are ribonucleoprotein (RNP) granules that form through dynamic assembly of RNAs and interacting proteins. Disease-associated RBPs such as hnRNPA1 [243], hnRNPA2/B1 [244], TIA1 [61], Matrin3 [49], annexin A11 [113], FUS [245] and TDP-43 [166] contain a LCD and have been proposed to undergo de-mixing in membraneless organelles through LLPS [228, 229] (Fig. 4A). Notably, a genetic link with ALS and/or FTD has been identified for each of the aforementioned phase-separating RBPs [246], highlighting dysfunctional assembly of RBP-containing condensates as a potential key pathogenic perturbation.

The phase separation behavior of TDP-43 and FUS has been particularly well studied. Indeed, purified full-length TDP-43 or TDP-43 LCD can de-mix into dynamic condensates in cell-free assays [164, 165, 167, 169, 247–253], and TDP-43 undergoes LLPS in the nucleus, cell body and axonal compartment of neurons [167, 223, 254–260]. Likewise, FUS phase separates in cell-free in vitro systems and in cells [225, 245, 261–266]. Although intrinsically disordered domains are a common feature among phase separating proteins [243, 267–269], structured proteins without LCDs have also been reported to undergo LLPS [270], while interaction of disordered proteins can occur in the absence of LLPS [271]. Phase separation behavior of RBPs is ultimately driven by the formation of a multivalent network, supported by multivalent interactions among LCDs, as well as interactions between folded protein domains, protein-RNA interactions and RNA-RNA interactions. For a more in-depth description of the protein sequences and interactions that underlie phase separation, we refer readers to the following reviews [272–274].

ALS/FTD-linked RBPs serve as components and/or regulate the formation of several types of functional biomolecular condensates in the cell. For instance, endogenous TDP-43 and/or FUS have been described to localize to and regulate the formation of 1) *paraspeckles*, which are nuclear membraneless bodies characterized by the presence of the long non-coding RNA NEAT1 [215, 275–277], 2) *Cajal bodies*, another class of nuclear RNP granules which act as platforms for the assembly and modification of the spliceosome complex [278], but also 3) *stress granules*, which are RNA–protein assemblies that form in the cytoplasm as a protective mechanism against a variety of cellular stressors [211]. Interestingly not only TDP-43 [211, 279, 280] and FUS [211, 281], but also TAF15 [282], hnRNPA1 [283], hnRNPA2B1 [284], and TIA1 [285] have been involved in their regulation. Finally 4) *transport granules* are another example of RNA-containing biomolecular condensates of particular relevance in neuronal cells as they enable transport of mRNA transcripts from the soma, along axons and dendrites, to distal neuronal compartments for their local translation [37, 286]. TDP-43 – when overexpressed with a fluorescent tag in fly, mouse or rat neurons – localizes to these transport granules, and ALS-associated TDP-43 mutations impair their dynamic properties and consequently disrupt axonal transport kinetics [208, 223]. FUS has also been proposed to facilitate mRNA transport along dendrites from cultured hippocampal neurons [209]. Interestingly, a recent study demonstrated that a subset of axonal RNA granules “hitchhike” on moving lysosomes, a process facilitated by annexin A11. The LCD of annexin A11 mediates its de-mixing with RNA granules through LLPS, while its membrane-binding domain interacts with lysosomes effectively acting as a molecular tether between these two organelles [113].

Through their LLPS properties, TDP-43 and FUS regulate a wide range of RNA metabolic processes. It was recently reported that endogenous expression of LLPS-deficient TDP-43 in mice [164] results in impaired neuronal function and translation defects (without detectable TDP-43 pathology or neurodegeneration) [287]. In addition, several independent studies demonstrated that TDP-43 condensation is required for its binding to specific RNA regions across the transcriptome, and that ectopic expression of LLPS-deficient TDP-43 mutants in turn results in impaired RNA processing functions such as TDP-43-mediated autoregulation [152, 165, 167, 252]. In contrast, another study suggested that LLPS-impaired TDP-43 does still support splicing of other RNA targets [256], underscoring that the effects of TDP-43 condensation on splicing might be specific for a subset of RNA binding sequences [167]. Additional work is needed to further elucidate the contribution of TDP-43 LLPS in the context of RNA splicing including in vivo.

#### Aberrant phase transitions in disease

While condensation of TDP-43 and FUS via LLPS is indispensable for their physiological functions, aberrant phase transitions can convert dynamic condensates to gel- or solid-like assemblies, suggesting that dysregulated LLPS might play a role in the initiation of their pathological aggregation in neurodegenerative diseases [228–230] (Fig. 4A). This “liquid-to-solid phase transition” hypothesis is supported by a large number of studies that report aberrant dynamic properties of TDP-43 [164, 253–255] and FUS [225, 245, 261] in the presence of disease mutations, ageing or other stressors [166, 254]. Conversion of liquid-like condensates into gel- or solid-like assemblies has also been observed for other ALS/FTD-linked RBPs, including hnRNPA1 [243, 269], hnRNPA2 [244] and TIA1 [288]. Regardless, the precise origin of the insoluble inclusions that accumulate in ALS and FTD remains heavily debated.

Stress granules (SGs) protect cells (of the nervous system) against transient stress, but have also been proposed to serve as a potential link between physiological condensates and pathological aggregates [211, 289–291]. For example, exposure to a variety of stressors was reported to provoke accumulation of phosphorylated TDP-43 within SGs in cancer cell lines, neuronal cultures and ALS patient fibroblasts [251, 292–295]. ALS/FTD-associated mutations in the essential SG protein TIA1 resulted in accumulation of SGs with aberrant recovery dynamics, further accompanied by recruitment of insoluble endogenous TDP-43 in HeLa cells [61]. SGs artificially generated by light-induced multimerization of the SG scaffold protein G3BP1 were also reported to eventually accumulate endogenous TDP-43 along with pathological markers such as ubiquitin and p62 in cancer cell lines [296].

On the other hand, mounting evidence also reveal that pathological inclusions can initiate independently of SGs. Indeed, Gasset-Rosa and colleagues demonstrated that either induced-expression of TDP-43 in the cytoplasm – or cytoplasmic recruitment of endogenous TDP-43 by exposure to amyloid-like recombinant aggregates – results in the accumulation of cytoplasmic TDP-43 condensates by LLPS, with no recruitment of SG markers [254]. Upon further transient exposure to arsenite, these cytoplasmic TDP-43 condensates transition to a gel/solid-like state which induces nuclear transport defects and eventually cell death. Using an independent approach relying on optogenetics to induce light-dependent multimerization of TDP-43 in the cytoplasm, Mann and colleagues produced insoluble TDP-43 assemblies that co-localize with pathological markers (such as phosphorylated TDP-43 and p62) but without recruiting SG components [255]. This was also associated with toxicity in cultured cells [255] and *Drosophila* models [297]. More recently, using live-cell single molecule tracking microscopy, Streit et al. reported that mobility of overexpressed fluorescently-labeled TDP-43 in cultured cell lines was reduced in SGs – but also in several other “diffuse” TDP-43 patches throughout the cytoplasm – in response to different cellular stressors [259], further providing direct evidence that aggregation of RBPs can initiate independently of SG compartments. While early studies reported overlap of SG markers with TDP-43 inclusions in the ALS or FTLD-TDP nervous system [293, 294], a more recent analysis of late-stage ALS patient spinal cords showed that SG components were predominantly excluded from TDP-43 pathology in diseased motor neurons [298].

Multiple factors modulate the condensation behavior of RBPs, and their dysregulation might act as a trigger to initiate the accumulation of pathological aggregates in disease. For instance, biophysical properties of RNP condensates can be modified by genetic mutations or post-translational modifications within the RBPs [250, 264, 299], by interactions with RNA sequences [248, 300, 301], or by interaction with molecular chaperones such as heat shock proteins [257, 258, 262] or nuclear import receptors [174, 263–265]. A more in-depth description of the factors that regulate phase separation and phase transitions of TDP-43 and FUS has been recently reviewed [166].

## Prion-like propagation of RBP pathology in ALS and FTD

### Amyloid filaments in ALS and FTD

The abnormal aggregation of misfolded proteins in the CNS is a defining feature of most human neurodegenerative disorders [1]. These aggregating proteins typically assemble into amyloid filaments, which are highly structured fibrillar protein assemblies that are organized in a “cross-β sheet” motif (i.e. β-strands are stacked perpendicular to the fibril axis, and bound together by hydrogen bonds that extend along the fibril axis) [302, 303]. Recent developments in the field of structural biology have enabled to determine the atomic structures of these amyloid filaments, providing new opportunities for studying the corresponding neurodegenerative conditions (Tables 1 and 2).

**Table 1:** Amyloid structures of ALS/FTD patient-derived fibrils composed of RNA-binding proteins [84–86, 139]

**Study**	**Fibril source**	**Protein**	**Method**	**Amyloid structure**
**Arseni et al.** [84]	Extracts from frontal and motor cortex of patients with ALS and FTLD-TDP type B (n=2).	TDP-43	Cryo-EM	Amyloid-like fibril comprising a single protofilament (right-handed helical twist) with an ordered filament core (AA 282–360 in TDP-43 LCD) that adopts a “double-spiral shaped” fold.
**Arseni et al.** [85]	Extracts from prefrontal cortex of patients with FTLD-TDP type A (n=3).	TDP-43	Cryo-EM	Amyloid-like fibril comprising a single protofilament (right-handed helical twist) with an ordered filament core (AA 272–360 in TDP-43 LCD) that adopts a “chevron-like” fold.
**Arseni et al.** [86]	Extracts from prefrontal and temporal cortex of patients with FTLD-TDP type C (n=4).	TDP-43 + ANXA11	Cryo-EM	Heteromeric amyloid-like fibril comprising a single protofilament (left-handed helical twist) with an ordered filament core composed of TDP-43 (AA 282/284-345) and ANXA11 (AA 39-74) LCD regions.
**Tetter et al.** [139]	Extracts from prefrontal and temporal cortex (n=4), as well as motor cortex and brain stem (n=2), of FTLD-FUS/FTLD-FET patients (with motor neuron pathology).	TAF15	Cryo-EM	Amyloid-like fibril comprising a single protofilament (left-handed helical twist) with an ordered filament core composed of the LCD of TAF15 (AA 7-99).

**Table 2:** Amyloid structures of recombinant fibrils composed of RNA-binding proteins [169, 170, 304–315]

**Study**	**Fibril source**	**Protein**	**Method**	**Amyloid structure**
**Li et al.** [170]	In vitro generated fibrils from complete LCD of TDP-43 (AA 267–414, with N-terminal His-tag).	TDP-43	Cryo-EM	Amyloid-like fibril comprising a single protofilament (left-handed helical twist) with a large and tightly packed filament core composed of TDP-43 LCD (AA 276-414).
**Sharma et al.** [305]	In vitro generated fibrils from full-length TDP-43 (AA 1-414, with N-terminal His-tag).	TDP-43	Cryo-EM	Three distinct amyloid-like fibrils that differ in the number and relative arrangement of their protofilaments, but have the same amyloid core composed of a region from the TDP-43 LCD (AA 304-348).
**Kumar et al.** [304]	In vitro generated fibrils from core peptide (AA 279-360) in TDP-43 LCD.	TDP-43	Cryo-EM	Amyloid-like fibril comprising one or two protofilaments (left-handed helical twist) with an ordered filament core (AA 281–351) that adopts an “H-shaped” fold.
**Cao et al.** [306]	In vitro generated fibrils from two peptides corresponding to AA 311-360 (SegA) and AA 286-331 (SegB) of TDP-43 LCD.	TDP-43	Cryo-EM	TDP-43 SegA (AA 311-360) forms 3 polymorphic fibrils which differ in the number and symmetry of protofilaments, but share an ordered filament core (AA 312–346) that adopts a “dagger-shaped” fold. TDP-43 SegB (AA 286-331), harboring the ALS-linked mutation A315E, forms one amyloid fibril comprising four protofilaments with an ordered core (AA 288–319) that adopts an “R-shaped” fold.
**Guenther et al.** [169]	In vitrogenerated fibrils from 10 small peptide segments of TDP-43 LCD.	TDP-43	X-ray diffraction	Six peptide segments form stable steric zippers (characteristic of irreversible pathogenic amyloid fibrils), while four peptide segments form unstable LARKS (characteristic of reversible protein hydrogels).
**Murray et al.** [308]	In vitro generated fibrils from complete LCD of FUS (AA 2-214, with N-terminal His-tag).	FUS	Solid-state NMR	Amyloid-like fibril with an ordered filament core that is composed of a small segment from FUS LCD (AA 39-95).
**Lee et al.** [309]	In vitro generated fibrils from C-terminal LCD (AA 111-214) of FUS.	FUS	Cryo-EM	Amyloid-like fibril comprising a single protofilament (left-handed helical twist) with an ordered filament core composed of a small segment from FUS LCD (AA 112-150), which is different from the core of the complete FUS LCD fibrils.
**Sun et al.** [307]	In vitro generated fibrils from complete LCD of FUS (AA 2-214, fused to N-terminal His-mCerulean).	FUS	Cryo-EM	Amyloid-like fibril comprising a single protofilament (left-handed helical twist) with an ordered filament core composed of a segment from FUS LCD (AA 34-124), which is larger and more stable compared to previous FUS LCD fibrils.
**Luo et al.** [313]**;** **Hughes et al.** [314]	In vitrogenerated fibrils from small peptide segments of FUS LCD	FUS	X-ray diffraction	Highly unstable amyloid fibrils formed by “SYSGYS” and “SYSSYG” motifs which occur in the FUS LCD.
**Sun et al.** [310]	In vitro generated fibrils from complete LCD (AA 186-320) of hnRNPA1.	hnRNPA1	Cryo-EM	Amyloid-like fibril comprising two protofilaments (left-handed helical twist) with an ordered filament core composed of a small segment of the hnRNPA1 LCD (AA 251–295), which contains the hnRNPA1 PY-NLS motif (AA 263–289) that interacts with karyopherin β2.
**Sharma et al.** [311]	In vitro generated fibrils from full-length hnRNPA1 (AA 1-320).	hnRNPA1	Cryo-EM	Amyloid-like fibril comprising two protofilaments (left-handed helical twist) with an ordered filament core composed of a small segment of the hnRNPA1 LCD (AA 251–295). This amyloid structure is almost identical to the one formed by hnRNPA1 LCD [310].
**Lu et al.** [315]**; Lu et al.** [312]	In vitro generated fibrils from complete LCD of hnRNPA2 (AA 181-341, fused to N-terminal mCherry tag).	hnRNPA2	Cryo-EM	Amyloid-like fibril comprising one asymmetric protofilament (left-handed helical twist) with an ordered filament core composed of a segment from the hnRNPA2 LCD (AA 263–319), which encompasses the hnRNPA2 PY-NLS motif. The disease-associated D290V mutation generates three polymorphic hnRNPA2 LCD fibrils which are more stable than WT fibrils.

#### TDP-43

Several early studies demonstrated that end-stage aggregates in ALS or FTLD-TDP autopsies consist of amyloid-like TDP-43 fibrils with diameters of 10–15 nm [316–319]. More recently, cryo-electron microscopy (cryo-EM) has revealed the composition of patient-derived fibrils of TDP-43 at near atomic resolution [84–86, 302] (Table 1). This demonstrated that TDP-43 fibrils are single helical filaments with an ordered core that is made up of a segment from the LCD of TDP-43, while the N- and C-termini form a “fuzzy coat” around this filament core that cannot be structurally resolved. While the general structure of TDP-43 amyloid filaments is conserved across neurodegenerative diseases, the precise fold adopted by individual TDP-43 monomers within the core – and how they stack together to form these filaments – can differ between different TDP-43 proteinopathies. Arseni and colleagues demonstrated that TDP-43 amyloid filaments extracted from individuals with ALS or FTLD-TDP type B are characterized by a “double-spiral-shaped” fold, regardless of the brain region from which the aggregates were extracted [84]. On the other hand, TDP-43 is reported to adopt a different fold (termed a “chevron-like fold”) in fibrils of patients with FTLD-TDP type A [85]. In line with this, earlier studies indirectly evaluated the conformation of aggregated TDP-43 in different brain regions and spinal cord of ALS and FTLD-TDP by immunoblotting protease-resistant TDP-43 fragments from detergent-insoluble homogenates: distinct banding patterns of TDP-43 fragments were observed in different disease subtypes (presumably reflecting pathological TDP-43 species with different structures, also referred to as “strains”), while banding patterns were identical across multiple CNS regions in individual patients [320]. Moreover, cell-free in vitro studies demonstrated that recombinant TDP-43 and TDP-43 fragments can assemble to form amyloid-like filaments which acquire distinct amyloid folds depending on the experimental paradigm and protein region used for fibrillization [169, 170, 304–306] (Table 2). Hence, at an ultrastructural level, TDP-43 has the ability to form several different amyloid structures in vitro and in human disease. This is in line with amyloid structures for other disease proteins like tau and α-synuclein, where distinct amyloid folds also define different neurodegenerative conditions [302]. It has been suggested that these disease-specific amyloid structures could arise from differences in cellular environment, post-translational modifications or interaction partners in distinct brain cells, but the exact factors driving this structural heterogeneity remain to be elucidated [302].

An exciting recent development is the cryo-EM structure elucidation of filaments extracted from the brains of four individuals with FTLD-TDP type C [86]. Unexpectedly, this revealed that the ALS-associated RBP annexin A11 (ANXA11) co-assembles with TDP-43 in heteromeric FTLD-TDP type C amyloid filaments, whereby the ordered filament core is composed of defined regions from the LCDs of TDP-43 and ANXA11 respectively [86]. This is the first report of a patient-derived heterotypic amyloid filament for TDP-43, and it raises the question whether TDP-43 could also co-assemble with the LCD of other ALS-linked RBPs – such as hnRNPA1 or hnRNPA2B1 which have been shown to co-localize with pathological TDP-43 inclusions in patients that carry mutations in the corresponding genes [47]. It is noteworthy that multiple independent studies also identified abundant amyloid filaments composed of a C-terminal fragment of the lysosomal protein TMEM106B in the brains of patients with TDP-43 proteinopathies, tauopathies and synucleinopathies, but also in older neurologically normal individuals [321–323]. These findings are intriguing, given that genetic variants in the *TMEM106B* locus had previously been identified as a risk factor for FTLD-TDP [324, 325]. Whether and how TMEM106B amyloid filaments contribute to neurodegenerative diseases remains to be elucidated.

#### FUS and TAF15

FUS forms liquid-like condensates by LLPS, which can transition into fibrous structures that contain amyloid-like filaments upon ageing and disease-causing mutations [225, 245]. A number of studies have subsequently resolved the ultrastructure of recombinant FUS filaments, revealing an amyloid core composed of a segment from the FUS LCD [307–309] (Table 2). Likewise, peptide segments within the LCD of TAF15 are also capable of assembling into amyloid-like filaments in cell-free assays [326].

A recent study by Tetter and colleagues determined the ultrastructure of amyloid filaments extracted from the prefrontal and temporal cortex of four patients with FTLD-FUS/FTLD-FET [139] (Table 1), in which pathology is characterized by cytoplasmic inclusions of FUS and TAF15 [117]. Surprisingly, these filaments were composed of a segment from the LCD of TAF15 rather than FUS [139]. The reason that FUS-containing fibrils could not be identified in brain homogenates from FTLD-FUS/FTLD-FET is currently unclear. One possible explanation is that FUS filaments are much less abundant than TAF15 filaments, or could not be captured by the extraction method used in this study. Alternatively, FUS may be recruited to TAF15 amyloid aggregates in a non-filamentous aggregation state. Further work is thus needed to solve the structural nature of FUS aggregates in FTLD.

FUS-containing cytoplasmic inclusions also accumulate in ALS with *FUS* mutations, where FUS aggregation occurs in the absence of TAF15 pathology [30, 31]. It will therefore be important to characterize the structure of amyloid filaments that accumulate in the nervous system of these patients as well.

### Prion-like properties of RBPs in ALS and FTD

A subclass of amyloids that is of particular interest are prion proteins, in which aggregation occurs in a self-templating and infectious fashion [327]. The term “prion” (“proteinaceous infectious particle”) was coined in 1982 by Nobel Prize laureate Stanley Prusiner, who demonstrated that templated misfolding and aggregation of the prion protein PrP is the underlying cause of a class of infectious neurodegenerative disorders termed transmissible spongiform encephalopathies (now often denoted as “prion diseases”) [328]. In these disorders, the native prion protein (PrP^C^) can misfold and convert into a pathogenic prion protein conformer (PrP^Sc^), which subsequently acts as a template – or “seed” – by transferring this abnormal conformation to other surrounding prion proteins, further inducing their misfolding and aggregation. Moreover, these pathogenic seeds have been reported to be transferred between cells, resulting in progressive spreading of prion pathology throughout the nervous system [327]. Intriguingly, amyloid-like proteins that form aggregates in common age-related neurodegenerative diseases – such amyloid-β, tau, α-synuclein and more recently TDP-43 and FUS – have been proposed to display some properties that resemble prions (Fig. 4B) [329]. It is important to highlight that while some neurodegenerative diseases (e.g. AD, PD, ALS and FTD) can be classified as prion-like disorders due to proposed seeding and spreading of the underlying pathology, they are not considered infectious (in contrast to prion diseases) since there is no evidence of transmission between individuals.

#### TDP-43

Mounting evidence suggests that TDP-43 pathology propagates via a prion-like mechanism. Firstly, neuropathological analyses of autopsied CNS from ALS and FTLD-TDP patients point to spreading of TDP-43 pathology in a stereotypical manner from the site of initiation to anatomically connected brain regions [73, 89], further correlating with the propagation of neurodegeneration throughout the CNS [74]. Secondly, cryo-EM based characterization of TDP-43 fibrils extracted from different regions of a given brain reveals identical structures, as expected in case of templated aggregation [84]. This is in line with earlier studies which reported that immunoblotting of aggregated TDP-43 from autopsied brain and spinal cord material yields a unique pattern of protease-resistant TDP-43 fragments within an individual patient, regardless of the brain region from which the aggregates were extracted [320]. Thirdly, the LCD of TDP-43 bears a striking resemblance to yeast prion proteins in terms of its amino acid composition, and is therefore also termed “prion-like domain” [162, 163, 330, 331]. Moreover, recombinant TDP-43 LCD assembles into amyloid-like filaments that can accelerate aggregation of soluble TDP-43 in cell-free assays [247, 253]. A final argument in favor of prion-like propagation of TDP-43 pathology comes from experimental studies demonstrating that accumulation of insoluble TDP-43 can be directly induced in cultured cells [254, 304, 332–337] and transgenic mouse models [335, 338] through exposure to patient-derived brain extracts or fibrils generated from recombinant TDP-43.

One of the first reports suggesting that pathological aggregates in ALS and FTLD-TDP display prion-like properties in cells came from Nonaka and colleagues in 2013 [332]. Detergent-insoluble material from brains of individuals with ALS or FTLD-TDP introduced in a cultured cell line overexpressing TDP-43 revealed recruitment of the ectopically expressed TDP-43 into insoluble aggregates, and this was associated with TDP-43 phosphorylation and C-terminal truncation. This suggested that ALS/FTLD brain extracts may contain pathogenic material (presumably TDP-43 fibrils) that can seed aggregation of cellular TDP-43. These early findings have since been supported by numerous other efforts using cell lines overexpressing wild-type or mutant forms of TDP-43 [333–335, 338], human iPSC-derived motor neurons and astrocytes [339], or human iPSC-derived cerebral organoids [340].

A recent study by our team and collaborators demonstrated that fibrils produced from recombinant TDP-43 LCD also trigger TDP-43 pathology including both cytoplasmic aggregation and nuclear clearance, when introduced in human cell lines and iPSC-derived neurons [336]. These seeded TDP-43 aggregates rapidly acquired distinct biophysical properties and recapitulated disease hallmarks such as phosphorylation, ubiquitination and p62 accumulation. Importantly, fibril-induced cytoplasmic aggregation of TDP-43 was tightly accompanied by its depletion from the nucleus, resulting in a unique transcriptomic profile including disease-associated cryptic mRNA splicing [336]. An independent study by Scialò and colleagues obtained similar findings in SH-SY5Y cells [337]: recombinant TDP-43 LCD fibrils were readily internalized and transferred between cells, and recruited endogenous nuclear TDP-43 into cytoplasmic aggregates, resulting in a transcriptomic and proteomic signature associated with both TDP-43 cytoplasmic gain-of-toxicity and nuclear loss-of-function. Altogether, these findings further support a prion-like paradigm wherein TDP-43 aggregation occurs in a templated fashion, and suggest that templated TDP-43 aggregation in turn triggers TDP-43 nuclear clearance – hence accounting for the two major pathological hallmarks of TDP-43 proteinopathies [336, 337].

As discussed in Aggregation of RNA-binding proteins in ALS and FTD section, TDP-43 aggregates in ALS and different FTLD-TDP subtypes display significant morphological heterogeneity. Intriguingly, Smethurst and colleagues reported that ALS autopsied material also induces a mix of differently shaped TDP-43 inclusions when exposed to cultured cells that ectopically express TDP-43 [333]. In addition, two independent groups compared the prion-like seeding properties of FTLD-TDP brain extracts from different pathological subtypes, and found that each type of brain pathology induces TDP-43 aggregates with distinct morphologies in non-neuronal cell lines [334, 335]. Porta and colleagues found that FTLD-TDP type A and B brain samples induced spherical TDP-43 inclusions when transfected in cultured cells overexpressing cytoplasmic mutant TDP-43, while extracts from FTLD-TDP type E brains caused “skein-like” TDP-43 aggregates [335]. Similarly, De Rossi et al. reported that FTLD-TDP type A and type C brain extracts, respectively, displayed different seeding capacities in TDP-43 overexpressing human embryonic kidney (HEK) cells [334], and induced differential neurotoxicity upon exposure to neuronal cultures [81]. Altogether, the authors hypothesized that the different conformational forms of pathological TDP-43 that occur across different FTLD-TDP pathological subtypes – also referred to as strains, as discussed above – have distinct seeding properties and can induce aggregates with heterogeneous shapes in cells, albeit the underlying molecular mechanisms being incompletely understood. We recently demonstrated that in vitro-generated recombinant TDP-43 LCD fibrils with time also induce heterogeneous morphologies of fluorescently-labeled TDP-43 aggregates – including compacted, filamentous and fragmented – in U2OS cells and iPSC-derived neurons [336]. Surprisingly, these various aggregate morphologies were found to originate through progressive degradation of TDP-43 aggregates, and hence represent different stages of the degradation process. More work will be needed to determine whether similar mechanisms underlie pathological heterogeneity in patients with TDP-43 proteinopathies.

Brain-derived pathological TDP-43 assemblies can also seed aggregation of TDP-43 in vivo [335, 338]. Porta and colleagues reported that intracerebral injection of FTLD-TDP brain extracts into the CNS of transgenic mice, which inducibly overexpress cytoplasmic human TDP-43 in forebrain neurons, is accompanied by the formation of phosphorylated TDP-43 aggregates at the site of injection [338] – whereby induced expression of cytoplasmic mutant TDP-43 in itself was previously reported to provoke little TDP-43 pathology [341]. FTLD-TDP brain lysates were also reported to induce aggregation of wild-type mouse TDP-43 in non-transgenic mice, but pathology in these mice was much less pronounced [338]. Importantly, pathology of human cytoplasmic TDP-43 was proposed to spread throughout the mouse brain in a time-dependent fashion, and the injected brain extracts from patients with distinct pathological subtypes were associated with distinct spreading patterns [335]. More recently, a study reported that fibrils generated from recombinant TDP-43 induced phosphorylated TDP-43 accumulation when injected in the motor cortex of transgenic mice that overexpress human TDP-43 in the nervous system (under the Thy1 promoter), and this pathology was reported to progress to the bilateral motor cortex and lower motor neurons with time. The authors proposed that spreading may occur via pyramidal tract axons – which was associated with motor and cognitive dysfunction [342]. Further work is needed to understand how injection of brain homogenates in different CNS regions influences TDP-43 seeding/spreading, and to map potential spreading from brain to spinal cord and vice versa. It would also be of high interest to determine whether TDP-43 pathologies induced in vivo provoke neurodegeneration, and whether this is dependent on the pathological subtype.

Interestingly, Porta and colleagues also reported phosphorylated TDP-43 pathology within oligodendrocytes and astrocytes of transgenic mice injected with FTLD-TDP brain extracts, particularly at later timepoints, even though overexpression of cytoplasmic human TDP-43 in these animals is restricted to neurons [338]. Hence these findings could indicate that TDP-43 pathology may be transferred between neurons and glia, as is the case for pathology of other disease proteins such as tau and α-synuclein [343]. In line with this, a recent study reported that TDP-43 pathology in iPSC-derived motor neurons – induced by transfection of ALS spinal cord extract – could transfer to naïve iPSC-derived astrocytes (not exposed to ALS extract), and vice versa, when both cell types were co-cultured [339]. Moreover, astrocytes were suggested to reduce seeded TDP-43 aggregation and toxicity in motor neurons, suggesting a neuroprotective effect [339]. Besides astrocytes, a protective role from microglia in TDP-43 proteinopathies has also been reported. In mice that inducibly overexpress cytoplasmic human TDP-43, microglia were described to selectively clear TDP-43 from neurons and this was essential to restore motor function upon suppression of TDP-43 overexpression in these animals [344]. Interestingly, knock-out of *TREM2* – a genetic risk factor for AD – in mice that overexpress human TDP-43 has been shown to impair phagocytic clearance of TDP-43 by microglia [345]. Furthermore, progranulin-deficient microglia (in which the FTD gene *GRN* has been genetically deleted) were reported to directly promote the formation of cytoplasmic TDP-43 granules, along with nuclear pore defects and cell death, in cultured cortical neurons by secretion of complement factors in the extracellular medium [346]. Overall these findings highlight that different glial cell types may modulate and/or contribute to the amplification of pathological TDP-43 in the nervous system, yet more work is needed to further explore their precise role and define the underlying mechanisms.

The mechanisms underlying the prion-like spreading of TDP-43 pathology remain largely unresolved [347]. Exosomes/microvesicles have been proposed to be involved in the intercellular transmission of TDP-43 aggregates [332, 348–350]. Recent work also proposed a role for the cellular prion protein PrP^C^ in mediating direct neuronal uptake of TDP-43 seeds from the extracellular environment [351]. Nonetheless, alternative mechanisms could also be involved, including possibly TDP-43 spreading through direct contacts between cells or via tunneling nanotubes [348, 350, 352]. Interestingly, a recent study administered cerebrospinal fluid (CSF) from sporadic ALS patients or healthy control individuals into transgenic mice that overexpress human wild-type TDP-43 (hTDP-43) via chronic intracerebroventricular (ICV) infusion [353]. Treatment with CSF from ALS patients – but not control CSF – was reported to trigger cytoplasmic TDP-43 mislocalization in spinal motor neurons of hTDP-43 mice, along with increased levels of insoluble TDP-43. ALS-CSF treated animals displayed neuroinflammation, motor and cognitive dysfunction. Hence these findings suggest that CSF could provide a spreading route for pathology of TDP-43 proteinopathies [353]. In line with this, CSF samples from ALS and FTD patients could seed aggregation of recombinant TDP-43 LCD in cell free assays [354] – known as real-time quaking-induced conversion reaction (RT-QuIC) assays in which the amyloid dye Thioflavin T is used to detect amplification of prion-like proteins [355]. These data provide further evidence that ALS/FTD CSF samples may contain pathological TDP-43 with prion-like seeding properties.

While amplification of TDP-43 aggregation has mainly been studied in the context of the nervous system, it was recently suggested that insoluble TDP-43 in muscle tissue of mouse models and patients with inclusion body myopathy – a degenerative muscle disease with TDP-43 pathology – can seed TDP-43 oligomerization/aggregation in a FRET-based biosensor cell line, further highlighting that pathological TDP-43 in muscle tissue may also propagate in a self-templating fashion [356]. Consistently, an earlier study found that TDP-43 forms amyloid-like cytoplasmic assemblies in muscle tissue known as myogranules, which are crucial for normal muscle development and regeneration [226]. Intriguingly, these myogranules were reported to seed aggregation of recombinant TDP-43 in cell-free assays [226]. These findings indicate that prion-like aggregation of TDP-43 may not be restricted to the nervous system. Notably, in the context of synucleinopathies, intramuscular injection of recombinant α-synuclein fibrils was reported to trigger α-synuclein inclusion pathology in the brain and spinal cord of transgenic mice [357] – suggesting prion-like spreading of pathology from muscle to CNS. It is currently unclear whether such peripheral spreading routes also contribute to dissemination of TDP-43 pathology.

In summary, end-stage aggregates in brain or spinal cord (and potentially other tissue) autopsies of TDP-43 proteinopathy patients contain pathological TDP-43 – typically organized in amyloid-like filaments with distinct ultrastructures – and have been reported to seed aggregation and spreading of TDP-43 in cell culture models and transgenic mice via a prion-like cascade. It is noteworthy that TDP-43 proteinopathies often present as mixed pathologies in which aggregates of TDP-43 co-occur with aggregates of other amyloid-like proteins [100]. Because of this, several studies have investigated the potential interplay between these different disease proteins. For instance, the presence of TDP-43 pathology in post-mortem AD brains is found to be associated with increased burdens of tau pathology, and extracts of AD brains with TDP-43 pathology induce more pronounced tau aggregation in cellular seeding assays [358]. The importance of heterotypic protein aggregation in TDP-43 proteinopathies is further supported by the co-assembly of TDP-43 with ANXA11 in FTLD-TDP type C brains [86]. Furthermore, hnRNPA1 and hnRNPA2B1 have also been shown to assemble in amyloid-like filaments [47, 310–312, 315], and disease-associated mutations induce their co-localization with TDP-43 inclusions in affected tissues [47]. Determining whether and how co-aggregating proteins – such as ANXA11, hnRNPA1, hnRNPA2B1 or TMEM106B [47, 321–323] – can influence templated TDP-43 aggregation will provide valuable insights into underlying pathological mechanisms, and could help identify potential disease modifiers.

#### FUS and TAF15

Emerging evidence suggests that FUS pathology might propagate in a prion-like fashion. In an early in vitro study, Nomura and colleagues generated fibrils from recombinant FUS with an ALS-associated mutation, and reported that these mutant fibrils can serve as seeds to induce aggregation of monomeric wild-type FUS in a cell-free assay [359]. Recently, Vázquez-Sánchez and colleagues provided the first in vivo evidence in support of prion-like propagation of FUS pathology [360]. Indeed, amyloid-like fibrils produced from recombinant human FUS were injected into brains of ‘’humanized’’ mice that only express human wild-type or ALS-mutant FUS at endogenous levels, which triggered mislocalization and aggregation of endogenous human FUS in the mouse brain [360]. Importantly, fibril-induced FUS pathology spread from the site of injection to more distal brain regions in a time-dependent fashion, being more pronounced in the presence of ALS-associated *FUS* mutations, and ultimately exacerbated age-dependent cognitive and behavioral deficits from mutant human FUS expression. Interestingly, human FUS fibrils did not induce aggregation/spreading of wild-type mouse FUS in naïve mouse brains, indicating the existence of a species barrier. Cytoplasmic human FUS inclusions also recruited ubiquitin and p62 – but not TDP-43 – and were associated with increased levels of insoluble FUS and TAF15, further recapitulating FUS/TAF15 co-pathology as observed in FTLD [117]. These findings, along with the recent discovery of TAF15 amyloid filaments in the brain of FTD patients [139], highlight the need for studies that investigate prion-like seeding behavior of TAF15. In this respect, it will be of high interest to assess whether TAF15 fibrils can seed aggregation of TAF15 and/or FUS in cell and animal models, and further investigate whether genetic depletion of TAF15 can prevent pathological spreading of FUS and vice versa. Further work is thus eagerly anticipated to characterize the prion-like aggregation behavior of FUS and TAF15, and elucidate the molecular mechanisms that underlie the propagation and spreading of this pathology.

## Disease mechanisms downstream of RBP pathology

**Fig. 5:**
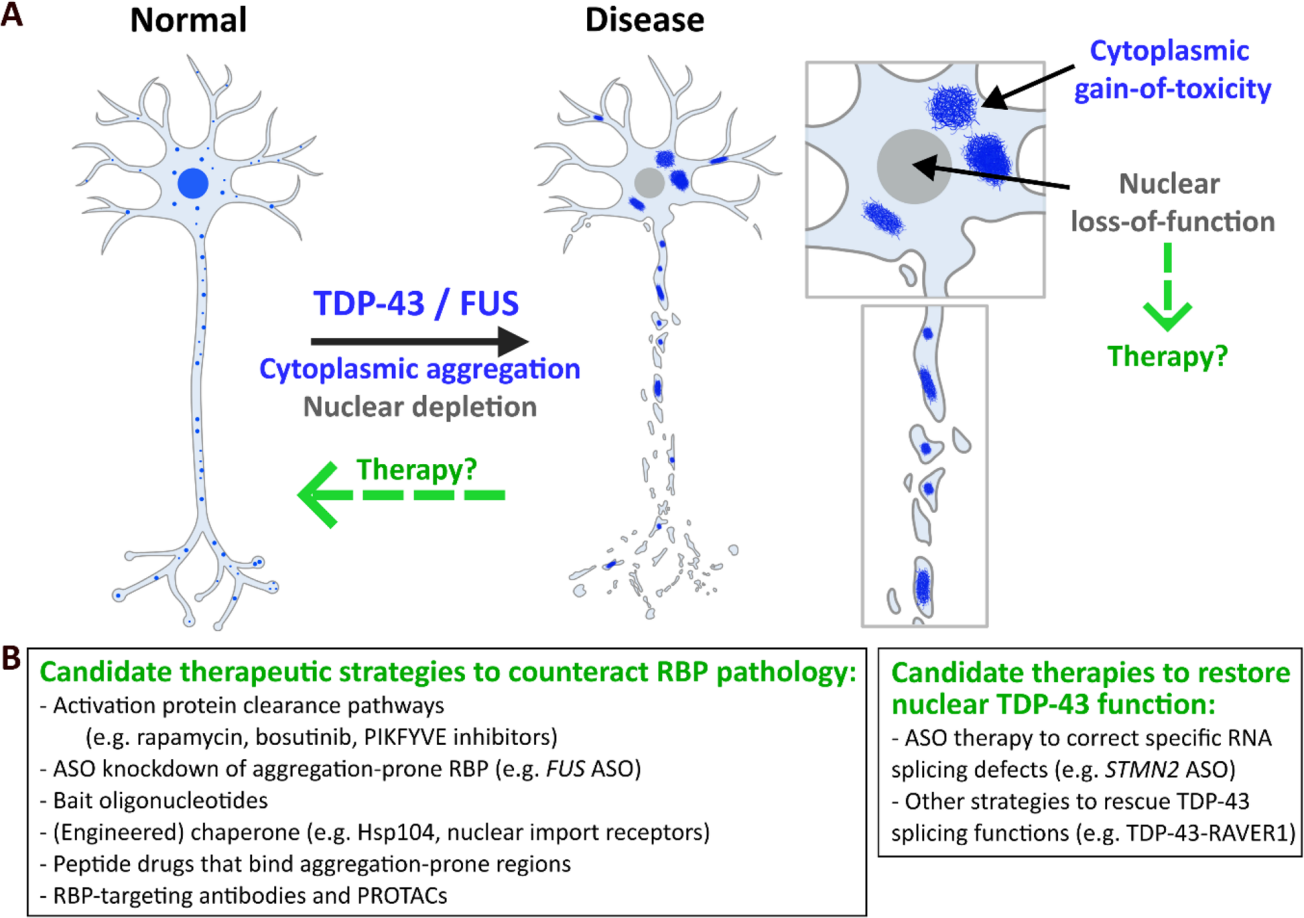
Gain-of-toxicity versus loss-of-function mechanisms in the pathogenesis of TDP-43 and FUS proteinopathies. **A** TDP-43 and FUS nuclear depletion and cytoplasmic aggregation in disease. RBPs are shown in blue. **B** Examples of candidate therapeutic strategies under investigation for treatment of TDP-43 and FUS proteinopathies, which aim to counteract TDP-43/FUS pathology or restore TDP-43 function in the nucleus

TDP-43 or FUS pathology is characterized by nuclear depletion and cytoplasmic aggregation in neurons and glia [36], although in the context of FUS the nuclear depletion is not consistent in all cases [36]. Hence, it has been proposed that TDP-43 and FUS proteinopathies could either originate from a loss-of-function mechanism (resulting from clearance of functional RBPs from the nucleus), a gain-of-toxicity mechanism (resulting from aberrant accumulation of pathological RBP aggregates in the cytoplasm), or a combination of both (Fig. 5A).

### Disease mechanisms downstream of TDP-43 pathology

TDP-43 is a key regulator of RNA metabolism, and its (nuclear) loss-of-function results in destabilization/dysregulation of thousands of mRNA transcripts, many of which are important for neuronal homeostasis [178–180, 196]. Accordingly, TDP-43 is an essential protein for CNS development [361, 362], and its selective depletion from motor neurons in the spinal cord causes neurodegeneration and motor phenotypes in mice [363]. In addition, expression of TDP-43 with ALS-causing mutations in transgenic mice was shown to induce TDP-43-dependent RNA splicing defects with age-related motor neuron degeneration, without detectable cytoplasmic TDP-43 aggregation (or nuclear TDP-43 depletion) [364], further suggesting that TDP-43-associated RNA splicing defects may suffice to drive neurodegeneration in animal models. It is noteworthy that these mice display splicing defects without loss of TDP-43 from the corresponding nuclei, while RNA splicing deficits in human TDP-43 disease typically result from nuclear depletion of TDP-43.

One TDP-43 target that is of particular relevance is stathmin-2 (*STMN2*), a highly abundant protein in neurons that is indispensable for the maintenance of axonal integrity [191, 192]. Multiple groups identified that stathmin-2 is incorrectly spliced (cryptic exon inclusion) and strongly downregulated upon TDP-43 depletion, and restoring its expression levels is sufficient to rescue axonal regeneration in TDP-43-depleted human neurons [184, 190]. In addition, TDP-43 regulates RNA processing of numerous other targets with proposed roles in neuronal function or development. This includes essential synaptic proteins such as NPTX2 [365], NOS1AP [366], AGRN [186] and UNC13 A [182, 193]. Interestingly, single nucleotide polymorphisms (SNPs) in *UNC13 A* are also among the strongest risk factors for ALS and FTD [367]. More recently, TDP-43 was also reported to interact with the lncRNA *neuroLNC*, and this interaction is essential to regulate synaptic vesicle release in rat neurons [368]. Furthermore, several studies reported TDP-43 targets that – upon dysregulation in the context of TDP-43 loss – could in turn promote gain-of-toxicity effects. For instance, TDP-43 regulates RNA splicing of hnRNPA1, and depletion of TDP-43 reportedly promotes hnRNPA1 aggregation by inducing accumulation of an alternatively spliced isoform with increased aggregation propensity [194]. In addition, TDP-43 regulates RNA processing of the SG assembly protein G3BP1 [369] and the essential autophagy protein ATG4B [186] – misprocessing of these transcripts has been hypothesized to impair the SG and autophagy response in disease affected cells with nuclear TDP-43 depletion. Finally, TDP-43 regulates alternative splicing of *MAPT* mRNA (encoding tau), and TDP-43 dysfunction alters the 3R-tau/4R-tau ratio in cultured cell lines and transgenic TDP-43 mice [370]. This could potentially contribute to the formation of neurofibrillary tangles in tauopathies, although further work is needed to test whether this is the case. In conclusion, TDP-43 loss-of-function-driven RNA dysregulation can potentially induce neurodegeneration by impairing multiple different pathways that are important for neuronal homeostasis.

Besides its role as a regulator of RNA metabolism, TDP-43 is reported to also have important functions related to its DNA-binding properties including in genome integrity: TDP-43 is thought to be involved in (1) the detection of DNA double-strand breaks as well as their repair by non-homologous end-joining (NHEJ) [371–373], (2) the assembly of DNA-repair complexes [374], and (3) the repair of R-loop associated DNA damage [373, 375, 376]. Accordingly, several studies report that loss of TDP-43, as well as the presence of ALS-associated TDP-43 mutations, induce DNA damage in cell lines, neuronal cultures, transgenic mouse models, and ALS patient tissues [371–376] – and the resulting genome instability has been proposed as an additional driver of neurodegeneration in TDP-43 proteinopathies. A more detailed description of the role of TDP-43 and FUS in the maintenance of DNA integrity and contribution to disease can be found elsewhere [222].

Several experimental paradigms have been attempted to recapitulate cytoplasmic accumulation of (ectopically expressed) TDP-43 in model systems – such as treatment with various cellular stressors, overexpression of wild-type or mutant TDP-43, ectopic expression of C-terminal TDP-43 fragments, or exposure to extracts containing pathogenic prion-like seeds [377]. This wealth of work suggests that accumulation of TDP-43 in the cytoplasm could induce cell death/neurodegeneration as well, via a toxic gain-of-function mechanism. For instance, inducible ectopic expression of a cytoplasmic form of human TDP-43 results in insoluble TDP-43 accumulation, neurodegeneration and motor impairments in transgenic mice [378]. Chronic cerebral hypoperfusion was recently shown to induce cytoplasmic accumulation of phosphorylated insoluble TDP-43 in cortical neurons of mice, and this was associated with cognitive and motor impairments [379]. Human TDP-43 overexpression also induces cell death in yeast and *Drosophila* [62], and a high-throughput genetic screening for modifiers of TDP-43-driven gain-of-toxicity in these models resulted in the identification of ataxin-2, one of the strongest risk genes for ALS in humans [62, 63]. It is noteworthy that expression of mutant TDP-43 at endogenous levels – in transgenic mice [364, 380–382] or cultured neurons [383, 384] – does typically not cause robust mislocalization and aggregation of TDP-43, suggesting that additional hits may be required for the development of TDP-43 pathology.

How cytoplasmic accumulation or aggregation of TDP-43 eventually drives disease has been widely studied and multiple pathogenic pathways have been proposed – the majority of which rely on overexpression of (wild-type/mutant/truncated) TDP-43 and/or treatment with (chemical or proteopathic) stressors [385]. For instance, TDP-43 aggregation can impair the integrity of proteostasis pathways. In support of this, multiple components of the ubiquitin–proteasome system (UPS) and autophagy pathway were reported to be sequestered within TDP-43 inclusions in ALS/FTD autopsies and disease models [386–390], which has been shown to impair the functionality of these protein quality control systems [390] – potentially initiating a vicious cycle that exacerbates TDP-43 pathology. Another proposed mechanism is TDP-43-induced disturbance in nucleocytoplasmic transport. Cytoplasmic TDP-43 condensates or aggregates can sequester components of the nuclear pore complex and nucleocytoplasmic transport machinery in cultured cell lines and neurons, mutant TDP-43 transgenic mice, and ALS-FTD postmortem CNS tissue [254, 391–396], which in turn could trigger deficits in nuclear transport of proteins such as TDP-43 itself. This is supported by recent findings identifying that nuclear pore injury in iPSC-derived neurons from ALS patients contributes to cytoplasmic mislocalization and dysfunction of TDP-43 [397–400]. Finally, emerging evidence suggests that accumulation of pathological TDP-43 in axons and synapses of neurons could contribute to neurodegeneration as well, which might be of particular importance since axonal degeneration and NMJ denervation are among the earliest ALS features [37, 76]. For instance, TDP-43-containing transport granules transition to a gel-like condensation state in the presence of ALS-associated TDP-43 mutations, which reportedly disrupts transport kinetics of RNP granules along axons [208, 223] (Liquid-liquid phase separation section). In addition, a recent study reported axonal accumulation of phosphorylated TDP-43 in iPSC-derived ALS motor neurons and a transgenic TDP-43 mouse model, and this was associated with toxicity due to suppressed protein synthesis in distal axons and NMJs [224].

### Disease mechanisms downstream of FUS pathology

As mentioned in Role in RNA metabolism section, loss of FUS results in aberrant processing of numerous RNAs involved in neuronal/synaptic maintenance and development. This includes transcripts encoding neurexins and neuroligins (i.e. synaptic adhesion molecules) [196], the AMPA receptor subunit GluA1 [401] and the dendritic spine maturation protein SynGAP [402]. As a result, global *FUS* knock-out in inbred mice causes perinatal lethality [403], highlighting its important role in development, and FUS depletion has been associated with synaptic defects in multiple in vitro and in vivo experimental model systems [401, 402, 404, 405]. Loss of FUS also promotes the pathological splicing of tau [406], and this has been suggested to contribute to accumulation of phosphorylated tau – along with neurodegeneration and cognitive defects – upon genetic silencing of FUS in the brain of mice [407]. Nevertheless, a number of studies have demonstrated that (conditional) knock-out of *FUS* is not sufficient to induce motor neuron degeneration or ALS-like phenotypes in mice [408, 409]. Hence, while FUS is important for neuronal function, nuclear depletion of FUS might not be the primary culprit in ALS-FUS.

In contrast, several independent studies demonstrated that progressive motor neuron degeneration and ALS-like phenotypes can be induced in mice by overexpressing human wild-type FUS [410, 411], expressing human FUS with ALS-associated mutations [210, 409, 412–414], or expressing cytoplasmic mutant FUS that lacks the NLS [415, 416]. In addition, genetic deletion of mouse FUS did not exacerbate motor phenotypes and neurotoxicity associated with expression of ALS-mutant human FUS in transgenic mice [210]. Hence, these findings are in favor of a toxic gain-of-function hypothesis, at least in the context of ALS-FUS. It is noteworthy that ALS-linked *FUS* mutations did not induce accumulation of FUS aggregates – even upon overexpression – suggesting that aggregation is not required to initiate disease at least in mice [210, 409, 412–414]. Yet, aggregation and spreading of FUS pathology contribute to the progression of FUS-mediated neurodegenerative disease: injection of recombinant FUS fibrils induced aggregation and spreading of mutant human FUS in the brain of transgenic mice, and this exacerbated age-dependent cognitive and behavioral deficits in these animals [360].

Several mechanisms have been proposed by which cytoplasmic FUS can exert toxicity and drive neurodegeneration, some of which are discussed here. In line with previously discussed findings for TDP-43, cytoplasmic accumulation of FUS has been shown to induce nucleocytoplasmic transport defects due to its interactions with nucleoporins, and this contributes to FUS induced toxicity in fly models [417]. Pathological FUS has also been shown to abnormally interact with other RBPs [418] and to trap snRNP components during spliceosome assembly [419]. FUS pathology in axons and synapses could also contribute to neurodegeneration. For instance, iPSC-derived motor neurons from ALS-FUS patients display cytoplasmic accumulation of FUS, and this is associated with hypo-excitability and defects in axonal transport [37, 420]. Moreover, cytoplasmic accumulation of mutant human FUS in cultured neurons has been shown to impair translation, including local translation in axons [37, 210, 225, 421, 422].

### Gain or loss of function?

Altogether, it is plausible that both mechanisms – loss-of-function in the nucleus and gain-of-toxicity in the cytoplasm – synergistically drive neurodegeneration in TDP-43 (and FUS) proteinopathies [423, 424] (Fig. 5). With this in mind, therapeutic strategies for TDP-43 proteinopathies should aim to (1) restore TDP-43 nuclear function, (2) remove cytoplasmic TDP-43 aggregates, or (3) prevent (prion-like) propagation of TDP-43 pathology; and ideally exert a combination of these effects. In contrast, the role of FUS loss-of-function in disease pathogenesis is not established, and most evidence indicates that FUS gain-of-function is likely the main driver of disease. Current therapeutic strategies under investigation for FUS proteinopathies are thus mainly focused on eliminating FUS pathology. It is important to highlight that the mechanisms by which RBP pathology drives toxicity might be different depending on the disease context. This is underscored by the different composition of FUS aggregates in ALS versus FTLD: FUS inclusions in FTLD are also positive for TAF15 (and EWS and transportin-1), while this is not the case in ALS-FUS [117]. Studies that further investigate the contribution of TAF15 in FTLD-FET pathogenesis will thus be of high importance. Likewise, the pathogenic role of RBPs that co-aggregate with TDP-43 – such as annexin A11 in FTLD-TDP type C – is currently poorly understood, which highlights the need to further investigate the interplay between different aggregation-prone RBPs and their relative contribution to disease.

## Therapeutic strategies to counteract RBP pathology

As outlined above, ALS and FTD are marked by mislocalization and aggregation of TDP-43 or FUS – at times in combination with other RBPs – and emerging evidence suggests that these protein pathologies propagate through the nervous system following a prion-like cascade, ultimately causing neurodegeneration via loss-of-function and/or toxic gain-of-function mechanisms. As a result, attractive therapeutic strategies for these conditions include approaches that restore normal RBP functions, or suppress the accumulation/spreading of toxic RBP species. Unfortunately, efforts to develop a cure or effective general treatment for all ALS and FTD forms have so far been unsuccessful, but several novel potential therapies are currently under investigation [425, 426] (Fig. 5B).

A first therapeutic strategy aims to correct downstream toxic mechanisms, rather than correcting RBP mislocalization/aggregation directly. One interesting example, specifically for treatment of TDP-43 proteinopathies, is ASO therapy to correct mis-processing of *STMN2* pre-mRNA which occurs as a result of nuclear loss of TDP-43 in disease. Preclinical studies provided proof-of-concept that ASOs can effectively restore normal *STMN2* splicing and rescue stathmin-2 neuronal functions in TDP-43 depleted neuron cultures and animal models [184, 192, 427]. A *STMN2*-targeting ASO, named QRL-201, is currently tested in human ALS patients to evaluate its safety and tolerability in a phase 1 clinical trial (NCT05633459). Despite promising preclinical evidence, it is important to note that *STMN2* is only one of many TDP-43 targets, and it remains to be determined whether rescuing stathmin-2 expression by itself will be sufficient to halt/delay disease progression in human patients. To overcome this limitation, a number of preclinical studies are investigating alternative approaches that aim to correct TDP-43 loss-of-function-driven RNA splicing defects in a more general fashion. An interesting strategy relies on a less aggregation-prone TDP-43 fusion protein named TDP-43-RAVER1 [186] – the N-terminal RNA-binding regions of TDP-43 fused to the general splicing repressor RAVER1 – that can be expressed in cells with TDP-43 pathology to complement for TDP-43 loss and restore TDP-43-mediated splicing functions [186, 428, 429]. These are promising approaches for precision therapy development in the context of TDP-43 proteinopathies.

An alternative therapeutic strategy aims to directly target pathology of TDP-43, FUS or other aggregation-prone proteins, by developing drugs that modify protein localization, aggregation or clearance. This includes several small molecules that affect general protein clearance pathways, which are tested in human ALS patients. Examples are ALS clinical trials with autophagy enhancers such as rapamycin [430] (NCT03359538) and bosutinib [431] (NCT04744532). Inhibition of the PIKFYVE kinase activates an unconventional protein clearance pathway in which aggregation-prone proteins are removed by exocytosis [432]. Pharmacological suppression of PIKFYVE kinase activity has been reported to ameliorate pathology in animal and iPSC-derived motor neuron models of familial and sporadic ALS [432], and a number of PIKFYVE inhibitors are currently also being tested in ALS clinical trials [433, 434].

An exciting advancement is the development of ASOs to specifically reduce levels of aggregation-prone proteins in the nervous system. In April 2023, the SOD1-targeting ASO drug Tofersen was approved by the U.S. Food and Drug Administration (FDA) after promising clinical trials in ALS patients with a mutation in the *SOD1* gene [435], providing a first proof-of-concept that this is a viable approach to reduce aggregation of pathogenic proteins in ALS. More recently, a FUS-targeting ASO (ION363) was developed for the treatment of ALS patients with *FUS* mutations. A preclinical study in transgenic mice with ALS-associated *FUS* mutations demonstrated that ICV injection of the FUS-ASO ION363 effectively reduced levels of FUS in the brain and spinal cord, which was sufficient to hamper motor neuron degeneration in these animal models [412]. In addition, ION363 was administered to a single human ALS patient with a FUS-P525L mutation by repeated intrathecal infusions, which provided the first preliminary evidence that this treatment can lower insoluble levels of wild-type and mutant FUS in the human nervous system [412]. Currently, clinical trials are ongoing to test the safety and efficacy of this ASO against FUS in larger cohorts of patients (NCT04768972). It is important to note that ASO therapy to lower total levels of TDP-43 are not considered a viable therapeutic strategy, given the detrimental consequences associated with TDP-43 loss [436]. Yet, allele-specific silencing of mutant TDP-43 might provide a potential therapeutic avenue for the treatment of familial ALS patients with *TARDBP* mutations.

An alternative treatment strategy for targeted reduction of mutant FUS exploits the autoregulatory feedback mechanism by which FUS regulates its own expression levels [221]. In a recent preclinical study, a wild-type *FUS* gene allele was introduced in a mouse model of ALS-FUS, which resulted in activation of an autoregulatory response and consequently reduced levels of mutant FUS in the cytoplasm. Importantly, wild-type FUS rescued lethality and motor phenotypes induced by cytoplasmic mutant FUS in these animals, underscoring the therapeutic potential of this approach [221]. Whether a similar strategy could be applied for reducing expression of mutant TDP-43 or other RBPs remains to be determined.

Multiple targeted approaches to modify the localization or aggregation of (wild-type) TDP-43 are currently being tested in preclinical studies [425]. Interesting examples include: the development of UG-rich “bait oligonucleotides” that specifically interact with TDP-43 to modulate its condensation and aggregation propensity [255], the investigation of (engineered) chaperones with disaggregation activity – such as nuclear import receptors [437] or yeast Hsp104 [438] – to counteract pathological TDP-43 aggregation, the development of peptide drugs that specifically interact with aggregation-prone regions in the TDP-43 LCD to suppress its aggregation [439], and the development of TDP-43-targeting antibodies [440–443] or small molecules (such as PROTACs) that specifically target aggregated TDP-43 for degradation [444]. It is important to note that strategies aimed at targeting aggregation-prone regions in the TDP-43 LCD are complex as it is critical to design approaches that specifically reduce aggregation without disrupting phase separation, given that the ability of TDP-43 to engage in LLPS is believed to be indispensable for its physiological functions.

Finally, future therapeutic strategies that aim to modify the prion-like propagation of pathological TDP-43 or FUS present another promising avenue for development of precision medicine in the context of TDP-43 or FUS proteinopathies. Such strategies could involve inhibiting initial aggregate seeding or blocking intercellular transmission pathways. An interesting example are therapeutic antibodies against tau which have been shown to inhibit seeding and propagation of tau pathology in transgenic mice exposed to AD brain extracts [445, 446]. Future studies investigating the potential of such therapeutic strategies to prevent amplification and propagation of RBP pathology in the context of ALS and FTD will be of high interest. Furthermore, novel TDP-43 and FUS seeding models (Prion-like properties of RBPs in ALS and FTD section) provide interesting opportunities to screen for drug candidates. Understanding the molecular determinants of prion-like behavior may also enable the development of biomarkers to monitor disease progression and therapeutic efficacy.

## Concluding statement

Aberrant condensation and aggregation of RBPs such as TDP-43 and FUS in the nervous system has emerged as a central theme in the pathogenesis of ALS and FTD, and mounting evidence highlights that RBP pathology can propagate in a fashion similar to prion proteins. While significant progress has been made in understanding the molecular underpinnings of RBP pathology, effective therapeutics remain elusive. Suppressing formation and/or spreading of pathological RBP assemblies, or restoring physiological RBP functions, may represent promising therapeutic strategies to halt/slow down disease. Physiological model systems that reliably recapitulate features of human pathology will be instrumental to translate relevant mechanistic insights into viable treatments to alter the course of ALS and FTD.

## Data Availability

Not applicable.

## Abbreviations

AD: Alzheimer's disease
AGRN: Agrin
ALS: Amyotrophic lateral sclerosis
ANXA11: Annexin A11
ASO: Antisense oligonucleotide
ATG4B: Autophagy related 4B
ATXN2: Ataxin-2
BIBD: Basophilic inclusion body disease
bvFTD: Behavioural variant FTD
C9ORF72: Chromosome 9 open reading frame locus 72
CLIP: Cross-linking and immunoprecipitation
CNS: Central nervous system
Cryo-EM: Cryo electron microscopy
CSF: Cerebrospinal fluid
DPR: Dipeptide repeat
EWS: Ewing's sarcoma protein
FDA: Food and drug administration
FRET: Förster resonance energy transfer
FTD: Frontotemporal dementia
FTLD: Frontotemporal lobar degeneration
FUS: Fused in sarcoma
G3BP1: G3BP Stress Granule Assembly Factor 1
GRN: Granulin precursor
HD: Huntington's disease
HEK: Human embryonic kidney
hnRNP: Heterogeneous nuclear ribonucleoprotein
HTT: Huntingtin
ICV: Intracerebroventricular
iPSC: Induced pluripotent stem cells
LATE: Limbic-predominant age-related TDP-43 encephalopathy
LCD: Low-complexity domain
LLPS: Liquid–liquid phase separation
MAPT: Microtubule-associated protein tau
MATR3: Matrin-3
NEAT1: Nuclear paraspeckle assembly transcript 1
NES: Nuclear export signal
NHEJ: Non-homologous end joining
NIFID: Neuronal intermediate filament inclusion disease
NIIBD: Neuronal intranuclear inclusion body disease
NLS: Nuclear localization signal
NMJ: Neuromuscular junction
NMR: Nuclear magnetic resonance
NPTX2: Neuronal Pentraxin 2
NTD: N-terminal domain
PD: Parkinson's disease
PPA: Primary progressive aphasia
PROTAC: Proteolysis targeting chimera
PrP: Prion protein
RAN: Repeat associated non-AUG
RBP: RNA-binding protein
RNA: Ribonucleic acid
RNP: Ribonucleoprotein
RRM: RNA-recognition motif
RT-QuIC: Real-time quaking-induced conversion
SC: Scarce cortical
SCA: Spinocerebellar ataxia
SETX: Senataxin
SG: Stress granule
SNP: Single nucleotide polymorphism
SOD1: Superoxide dismutase 1
STMN2: Stathmin 2
SynGAP: Synaptic Ras-GTPase activating protein 1
TAF15: TATA-binding protein-associated factor 15
TARDBP: Transactive response DNA-binding protein
TDP-43: Transactive response DNA-binding protein 43 kDa
TMEM106B: Transmembrane protein 106B
TREM2: Triggering receptor expressed on myeloid cells 2
UPS: Ubiquitin proteasome system
UTR: Untranslated region
VCP: Valosin-containing protein
ZnF: Zinc-finger
